# Staphylococcus aureus Uses the GraXRS Regulatory System To Sense and Adapt to the Acidified Phagolysosome in Macrophages

**DOI:** 10.1128/mBio.01143-18

**Published:** 2018-07-17

**Authors:** Ronald S. Flannagan, Robert C. Kuiack, Martin J. McGavin, David E. Heinrichs

**Affiliations:** a Department of Microbiology and Immunology, the University of Western Ontario, London, Ontario, Canada; New York University School of Medicine

**Keywords:** antimicrobial peptides, host-pathogen, intracellular bacteria, macrophages, phagocytosis, phagolysosome

## Abstract

Macrophages are critical to innate immunity due to their ability to phagocytose bacteria. The macrophage phagolysosome is a highly acidic organelle with potent antimicrobial properties, yet remarkably, ingested Staphylococcus aureus replicates within this niche. Herein we demonstrate that S. aureus requires the GraXRS regulatory system for growth within this niche, while the SaeRS and AgrAC two-component regulatory systems and the α-phenol soluble modulins are dispensable. Importantly, we find that it is exposure to acidic pH that is required for optimal growth of S. aureus inside fully acidified macrophage phagolysosomes. Exposure of S. aureus to acidic pH evokes GraS signaling, which in turn elicits an adaptive response that endows the bacteria with increased resistance to antimicrobial effectors, such as antimicrobial peptides, encountered inside macrophage phagolysosomes. Notably, pH-dependent induction of antimicrobial peptide resistance in S. aureus requires the GraS sensor kinase. GraS and MprF, a member of the GraS regulon, play an important role for bacterial survival in the acute stages of systemic infection, where in murine models of infection, S. aureus resides within liver-resident Kupffer cells. We conclude that GraXRS represents a vital regulatory system that functions to allow S. aureus to evade killing, prior to commencement of replication, within host antibacterial immune cells.

## IMPORTANCE

S. aureus can infect any site of the body, including the microbicidal phagolysosome of the macrophage. The ability of S. aureus to infect diverse niches necessitates that the bacteria be highly adaptable. Here we show that S. aureus responds to phagolysosome acidification to evoke changes in gene expression that enable the bacteria to resist phagolysosomal killing and to promote replication. Toxin production is dispensable for this response; however, the bacteria require the sensor kinase GraS, which transduces signals in response to acidic pH. GraS is necessary for phagolysosomal replication and survival of S. aureus in the acute stage of systemic infection. Disruption of this S. aureus adaptation would render S. aureus susceptible to phagocyte restriction.

## INTRODUCTION

Staphylococcus aureus is a notorious bacterial human and animal pathogen that is recognized as a serious threat to health care. Although S. aureus was once considered a nosocomial pathogen, it is evident that methicillin-resistant S. aureus (MRSA) strains, such as USA300 LAC, display enhanced virulence and can be disseminated throughout the community at large (1–3). Incredibly, S. aureus can colonize virtually every tissue of the body (4–7), and this propensity to cause infection can, in part, be attributed to the exceptional ability of S. aureus to circumvent host immunity. This is largely due to the vast repertoire of virulence determinants that S. aureus can express. A subset of these virulence factors are toxins (e.g., leucocidins) that poison host immune cells, such as macrophages and neutrophils, from the extracellular milieu, driven by the expression of host receptors (e.g., CD11b and CCR5) that are displayed at the plasmalemma of affected cells (reviewed in references 8 and 9). Despite this, neutrophils and macrophages are critical for host defense against S. aureus infection.

Professional phagocytes such as macrophages are central to innate immunity due to their ability to phagocytose particulates, including bacteria (10). Ingested microbes are internalized into membrane-bound vacuoles termed phagosomes that undergo maturation and become highly microbicidal organelles termed phagolysosomes (11). A hallmark of phagolysosome formation is the marked acidification of the phagosome lumen to a pH of ~5.4 or less due to the activity of the vacuolar ATPase (V-ATPase) proton pump (12, 13). Despite the antimicrobial activity of the phagolysosome, macrophages fail to eradicate internalized S. aureus cells (14–17). After phagocytosis of S. aureus USA300 by macrophages, the bacteria reside within mature lysosome-associated membrane protein-1 (LAMP-1)-positive phagolysosomes, where bacterial replication commences (15, 16). Interestingly growth of S. aureus within the macrophage is significantly delayed (15, 16). How S. aureus overcomes the antimicrobial aspects of the phagolysosome and grows in this niche is unknown. In the present study, we demonstrate that toxin production is dispensable for growth in the S. aureus-containing phagosome (SaCP). In contrast, we find phagolysosomal growth of S. aureus requires the GraXRS regulatory system. Moreover, through GraS, S. aureus can perceive phagolysosome acidification and elicit adaptive responses that engender phagolysosomal bacteria with the ability to resist killing and replicate. Finally, a murine model of systemic infection reveals that GraS is required for optimal survival in the acute stages of systemic infections when the bacteria reside within liver-resident Kupffer cells.

## RESULTS

### S. aureus growth in macrophages occurs independently of toxin production.

**FIG 1:**
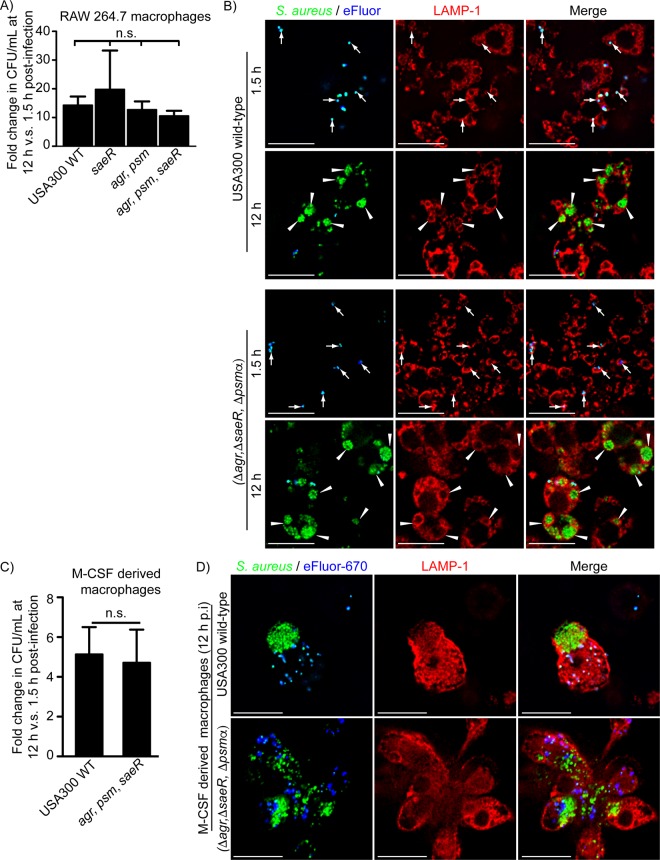
The two-component regulatory systems SaeRS and AgrAC and the PSMα peptides are dispensable for S. aureus growth in macrophages. In panel A, the graph depicts the fold change in CFU per milliliter at 12 h versus 1.5 h postinfection for wild-type S. aureus USA300 and mutant strains lacking a functional SaeR (*saeR*), Agr (*agr*), and the PSMα peptides (*psm*). The data are the mean ± standard error of the mean (SEM) from at least four independent experiments. n.s. indicates that differences in the data are not statistically significant (*P* ≥ 0.05) by one-way ANOVA with a Dunnett’s *post hoc* comparison. In panel B, representative images of fluorescence-based proliferation assays using RAW 264.7 macrophages and GFP-expressing wild-type S. aureus USA300 (top panels) and the triple mutant lacking the *sae*R, *agr*, and *psm*α genes (bottom panels) are shown. Endogenous LAMP-1 protein (in red) was detected by immunostaining macrophages at 1.5 and 12 h postinfection. GFP-expressing bacteria are green, and at the outset of the infection, all bacteria were labeled with eFluor-670 proliferation dye (in blue). At 12 h postinfection, replicating bacteria appear GFP positive yet far-red negative. The white arrows point to GFP- and eFluor-positive bacteria (i.e., cells that have not replicated). White arrowheads point to GFP-only bacteria that are demarcated by LAMP-1 and therefore growing inside late phagosomes/phagolysosomes inside RAW macrophages at 12 h postinfection. Bars equal ~10 µm. In panel C, the growth of S. aureus USA300 and the triple mutant inside primary human M-CSF-derived macrophages is summarized. The data are the mean ± SEM fold change in CFU per milliliter at 12 h versus 1.5 h postinfection for S. aureus USA300 and the triple mutant lacking *saeR*, *agr*, and the *psm*α genes. These data derive from 8 biological replicate infections using macrophages derived from four independent donors. n.s. indicates the difference between the means is not statistically significant by the Student’s unpaired *t* test (*P* ≥ 0.05). In panel D, representative micrographs depict wild-type S. aureus USA300 (top panels) and the triple mutant lacking *saeR*, *agr*, and the *psm*α genes (bottom) growing inside primary human M-CSF-derived macrophages at 12 h postinfection. Endogenous immunostained LAMP-1 protein is shown in red. Growing bacteria appear as GFP positive (green) yet proliferation dye (in blue) negative. Scale bars equal 10 µm.

In the extracellular environment, S. aureus elaborates toxins that poison immune cells such as macrophages. However, whether toxin production is important for the onset of S. aureus replication within the macrophage is unclear. Previous work has suggested that S. aureus may require either the Agr quorum sensing system or expression of alpha-toxin or alpha phenol soluble modulins (PSMα peptides) to survive and replicate within macrophages (18–20). Incongruent with these reports, it has been shown, both *in vitro* and *in vivo*, that S. aureus commences replication within macrophages while confined to acidic, LAMP-1-positive phagolysosomes (15, 16). Therefore, to further investigate the role of these genes and toxin production in the onset of S. aureus growth in the phagolysosome of macrophages, we constructed mutant strains of S. aureus USA300 lacking the entire *agrBDCA* locus (*agr*) and the *psm*α_*1–4*_ genes (*psm*α), a strain carrying a transposon-interrupted *saeR* gene (*saeR*::φNΣ), and a strain carrying all three mutations (*agr psm*α *sae*) (see Table 1 for descriptions). The *agr* and *sae* loci are global regulators of S. aureus virulence and are required for toxin production in S. aureus (21, 22). As expected, USA300 strains deficient for SaeR and/or Agr signaling demonstrated a lack of hemolytic activity on blood agar plates compared to wild-type (WT) S. aureus USA300 (see Fig. S1A in the supplemental material), consistent with previous reports (23, 24). Moreover, in contrast to wild-type bacteria, S. aureus strains deficient for *agr*, *psm*α, and *sae* also fail to intoxicate primary human macrophage colony-stimulating factor (M-CSF)-derived macrophages from the extracellular milieu, indicating a defect in toxin production (Fig. S1B). To assess whether these strains were indeed defective for growth within macrophages, gentamicin protection assays were performed using RAW 264.7 (here referred to as RAW) cells infected with wild-type S. aureus USA300 and each of the aforementioned mutants. This analysis revealed that at 12 h postinfection (hpi), each strain demonstrated at least a 9-fold increase in CFU per milliliter over that recovered for the same strain at 1.5 hpi (Fig. 1A). Moreover, there was no significant difference between wild-type S. aureus or the mutants in their ability to replicate within RAW macrophages (Fig. 1A). This was also evident when replication was investigated at the subcellular level by fluorescence microscopy. Here each strain expressing green fluorescent protein (GFP) was analyzed by fluorescence proliferation assays (see Materials and Methods for details) in conjunction with LAMP-1 immunofluorescence. In brief, just prior to infection, all GFP-expressing bacteria are colabeled with a far-red fluorescent proliferation dye, eFluor-670. As bacteria replicate, the dye is diluted until such time as the dye is no longer detectable on bacterial cells (i.e., replicating bacteria appear GFP positive yet proliferation dye negative). In contrast, bacteria that are unable to proliferate retain the proliferation dye and can thus be easily identified by fluorescence microscopy. This analysis revealed that at 1.5 hpi, none of the strains (i.e., USA300 or any of the mutants) had replicated, as all phagocytosed bacteria remained GFP and eFluor-670 positive (Fig. 1B). In contrast, by 12 hpi, replicating (i.e., GFP-positive, eFluor-670-negative) bacteria were observed within RAW macrophages for each strain (Fig. 1B; see Fig. S2 in the supplemental material). Moreover, immunodetection of LAMP-1 confirmed that wild-type and mutant bacteria are replicating within LAMP-1-positive phagosomes (Fig. 1B; Fig. S2). Importantly, there was no obvious difference in proliferation or LAMP-1 distribution around the S. aureus-containing phagosome (SaCP) for each strain. These observations in RAW cells were recapitulated in primary human M-CSF-derived macrophages; both WT and *agr sae psm*α bacteria produced a similar ~6-fold increase in bacterial burden within the 1.5- to 12-h time frame and appeared GFP positive yet eFluor negative when visualized within human M-CSF-derived macrophages (Fig. 1C and D). Taken together, these data reveal that S. aureus USA300 does not require *agr*, *sae*, *psm*α, or, by extension, toxin production to commence replication within LAMP-1-positive phagosomes in macrophages.

10.1128/mBio.01143-18.2 FIG S1  Analysis of toxin production in S. aureus *agr*, *psm*α, and *sae* mutants. In panel A, a single isolated colony of each of the indicated strains was streaked onto TSA plates supplemented with 5% (vol/vol) sheep blood. The plate was incubated at 37°C for 20 h and then incubated at 4°C for 6 h prior to being imaged. This blood agar plate is representative of two separate analyses. In panel B, the ability of S. aureus USA300 and a mutant lacking Agr and the PSMα peptides is shown. The panels show the presence of cells that are necrotic, as indicated by the nuclear accumulation of propidium iodide (PI [in red]) after 3 h of infection. In these experiments, extracellular bacteria were not killed with gentamicin and macrophages were infected at an MOI of 10. PI-stained cells were imaged live, and these images are representative of two-independent experiments using primary M-CSF-derived macrophages from two different donors. Download FIG S1, TIF file, 4.5 MB. Copyright © 2018 Flannagan et al. 2018 Flannagan et al. This content is distributed under the terms of the Creative Commons Attribution 4.0 International license.

10.1128/mBio.01143-18.3 FIG S2  Growth of wild-type S. aureus and toxin-deficient strains in macrophages. The representative fluorescent micrographs depict wild-type S. aureus USA300 and various toxin-deficient mutants replicating inside RAW macrophages at 12 h postinfection. Fluorescent proliferation assays reveal GFP-positive bacteria that are proliferation dye negative, indicating the bacteria have replicated. Endogenous LAMP-1 protein, detected by immunostaining, is shown in red. Scale bars equal 10 µm. Download FIG S2, TIF file, 4.5 MB. Copyright © 2018 Flannagan et al. 2018 Flannagan et al. This content is distributed under the terms of the Creative Commons Attribution 4.0 International license.

**TABLE 1:** Bacterial strains and plasmids used in this study

Bacterial strain or plasmid	Description^a^	Source or reference
Strains		
S. aureus		
USA300	USA300 LAC; hypervirulent community-associated MRSA; cured of antibiotic resistance plasmid	Laboratory stock
Newman	Wild-type clinical osteomyelitis isolate	55
RN4220	r_K_^−^ m_K_^+^; capable of accepting foreign DNA	56
*saeR* mutant	Derivative of S. aureus USA300 with *saeR*::φNΣ transduced from the Nebraska transposon library; Ery^r^	This study
*agr psm*α mutant	Derivative of S. aureus USA300 with Δ*psm*α_*1–4*_ and *agr*::*tetM*; Tet^r^	This study
*saeR agr psm*α mutant	Derivative of S. aureus USA300 with Δ*psm*α_*1–4*_ and *agr*::*tetM* and with *saeR*::φNΣ transduced from the Nebraska transposon mutant library; Tet^r^ Ery^r^	This study
*graS* mutant	Derivative of S. aureus USA300 with Δ*graS* created by pKOR mutagenesis	This study
*mprF* mutant	Derivative of S. aureus USA300 JE2 from the Nebraska transposon library carrying *mprF*::φNΣ; Ery^r^	
Newman *graS*	S. aureus strain Newman with *graS*::φNΣ allele transduced from the Nebraska transposon library; Ery^r^	This study
E. coli DH5α	F^−^ ϕ80d*lacZ*ΔM15 *recA1 endA1 gyrA96 thi*-*1 hsdR17*(r_K_^−^ m_K_^+^) *supE44 relA1* *deoR* Δ(*lacZYA-argF*)*U169 phoA* λ^−^	Laboratory stock

Plasmids		
pAH9	Constitutive staphylococcal mCherry expression vector; Ery^r^	57
P_*prsA*_::*gfp*	Constitutive S. aureus GFP expression vector; Amp^r^ Ery^r^	58
pCG44	E. coli/S. aureus shuttle vector for constitutive expression of pHluorin in S. aureus; Amp^r^ Cm^r^	59
pKOR	E. coli/S. aureus shuttle vector for creation of unmarked gene deletions in staphylococcal spp.; Amp^r^ Cm^r^	51
pKOR-GraS	pKOR with regions of homology to delete *graS*	This study
pKOR-αPSM	pKOR with regions of homology to delete *psm*α_*1–4*_	This study
pALC2073	E. coli/S. aureus shuttle vector; Amp^r^ Cm^r^	60
pGraS	pALC2073 with *graS* from S. aureus USA300	This study
pGYLux	Promoterless bioluminescent reporter plasmid encoding *luxABCDE*; Amp^r^ Cm^r^	61
pGYLux::*mprF*	pGYLux with the *mprF* promoter cloned	This study

^a^ Ery^r^, Tet^r^, Amp^r^, and Cm^r^ indicate resistance to erythromycin, tetracycline, ampicillin, and chloramphenicol, respectively.

### Replicating S. aureus bacteria reside within fully acidified phagolysosomes.

**FIG 2:**
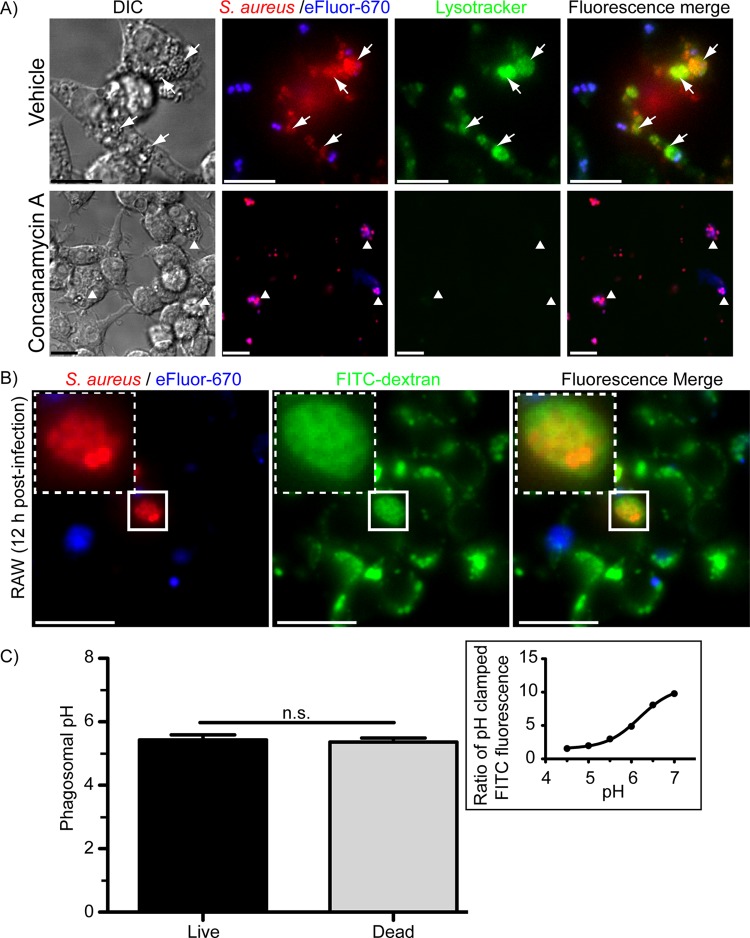
S. aureus USA300 replicates inside acidic phagolysosomes. In panel A, the acidity and integrity of the phagolysosomal membrane containing S. aureus were assessed by LysoTracker Green DND-26 staining. Macrophages were infected with live S. aureus USA300 cells expressing mCherry and labeled with eFluor proliferation dye. At 11 h postinfection, macrophages were left untreated or were treated with 1 µM ConA, and at 12 h postinfection, macrophages were stained with the acidotropic probe LysoTracker and imaged live. In the top panels, the white arrows point to replicating (i.e., eFluor-negative) bacteria that colocalize with LysoTracker Green fluorescence signal. In the bottom panel, the white arrowheads point to bacteria that are intracellular but now in the presence of ConA no longer colocalize with the acidotropic probe. Note not just phagosomes but also endosomes and lysosomes are also refractory to LysoTracker staining. These images are representative of at least three independent experiments. DIC, differential inference contrast. Bars equal ~10 µm. The residence of replicating S. aureus inside mature phagolysosomes (B) and pH of the compartment (C) in which these bacteria reside were established by dextran pulse-chase experiments. In panel B, a RAW macrophage loaded with 10,000-MW FITC-dextran and containing live replicating S. aureus USA300 at 12 h postinfection is shown. The white box delineates the region of the cell containing a large FITC-dextran-positive phagosome that also contains mCherry-positive yet eFluor-negative S. aureus USA300. In panel C, ratiometric pH measurements were made of FITC-dextran fluorescence (excitation 434 nm/excitation 490 nm) to quantify the pH of RAW cell phagolysosomes containing live and dead S. aureus USA300 cells. The graph shows the average pH ± standard deviation (SD) for live and dead S. aureus USA300 cells at 12 h postinfection. These data are the result of three independent experiments. n.s. indicates not significant as determined by Student’s *t* test. The representative graph shown in the inset reveals the pH responsiveness of FITC fluorescence as determined by pH clamping of live macrophages in K^+^-rich buffers with 10 µM nigericin.

Since replication of S. aureus in macrophages occurs after a significant delay (~10 to 12 h) (15, 16), we next sought to establish whether, when replicating intracellularly, S. aureus resides within acidified vacuoles, or whether the bacteria have modified the pH of the SaCP. To this end, fluorescent proliferation assays were performed on RAW macrophages infected with mCherry-expressing S. aureus cells and also stained with the acidotropic dye LysoTracker Green. Live-cell imaging revealed that at 12 h postinfection, well-defined vacuoles containing replicating S. aureus cells accumulate LysoTracker probe. In contrast, pretreatment of RAW macrophages with the V-ATPase inhibitor concanamycin A (ConA) renders infected macrophages completely refractory to LysoTracker staining, indicating that acidification of the SaCP is due to V-ATPase function (Fig. 2A). Moreover, these observations demonstrate that S. aureus can commence replicating within intact vacuoles that are acidic within murine macrophages.

To establish that replicating S. aureus is indeed inside mature phagolysosomes, we next performed pulse-chase experiments with fluorescein isothiocyanate (FITC)-dextran (25). Macrophages loaded with lysosomal dextran were infected with live or dead S. aureus cells, and at ~12 h postinfection macrophages were imaged by live-cell fluorescence microscopy. Previously we demonstrated that 30 min after engulfment, S. aureus colocalized with lysosomal dextran (15); however, here we show that even at 12 h postinfection, live and dead bacteria can remain colocalized with FITC-dextran in both RAW and primary human macrophages (Fig. 2B; see Fig. S3 in the supplemental material). Next, we performed quantitative measurement of phagolysosomal pH at 12 h postinfection by performing ratiometric pH measurements using FITC-dextran that is colocalized with intracellular S. aureus. These measurements revealed that the luminal pH of the SaCP harboring replicating and nonreplicating bacteria is on average 5.43 ± 0.16, which is comparable to the measured pH (5.36 ± 0.13) of phagolysosomes containing dead bacteria (Fig. 2C). The utility of our FITC-dextran to measure dynamic pH changes is evident from clamped dextran-loaded macrophages, where the fluorescence ratio of FITC, when excited at 490 and 430 nm, increases as cells are made more alkaline (Fig. 2C, inset). These data show that phagocytosed S. aureus cells can reside within intact phagolysosomes that are acidic, and this is where bacterial replication can commence.

10.1128/mBio.01143-18.4 FIG S3  In primary human M-CSF-derived macrophages, S. aureus colocalizes with lysosomal dextran long after phagocytosis. The images depict a primary human macrophage that was preloaded with FITC-dextran having phagocytosed mCherry-expressing S. aureus. At 12 h postinfection, the time images were taken, live S. aureus cells clearly colocalize with dextran, indicating the bacteria maintain their residence in a phagolysosome. This image is representative of at least two independent experiments with macrophages from different donors. Download FIG S3, TIF file, 1 MB. Copyright © 2018 Flannagan et al. 2018 Flannagan et al. This content is distributed under the terms of the Creative Commons Attribution 4.0 International license.

### The GraXRS regulatory system is required for growth within macrophage phagolysosomes.

**FIG 3:**
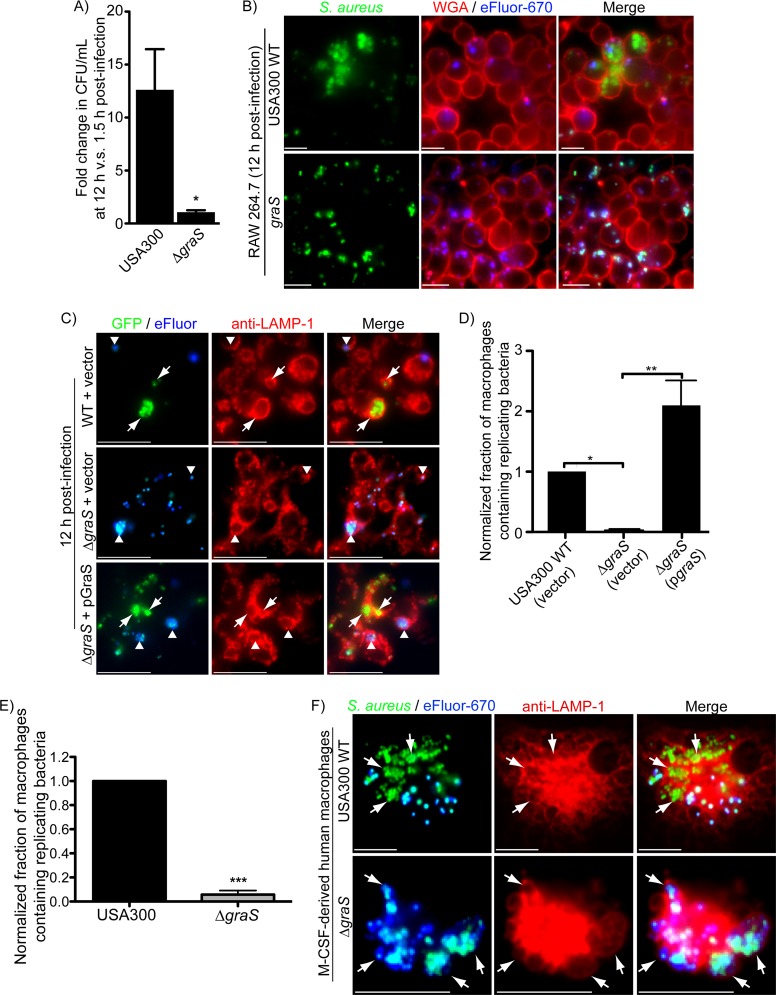
The sensor kinase GraS of the GraXRS regulatory system is required for S. aureus proliferation inside macrophages. The ability of S. aureus USA300 lacking the *graS* gene to grow inside RAW macrophages was compared to that of wild-type S. aureus USA300. In panel A, the fold change in CFU per milliliter at 12 h versus 1.5 h postinfection for wild-type USA300 and *graS* bacteria is shown. The data are the mean ± SEM derived from four independent experiments, and the asterisk indicates *P* ≤ 0.05. In panel B, the images depict the intracellular replication of wild-type S. aureus and the lack of replication for the *graS* mutant. Bacterial replication was determined by fluorescence proliferation assay, where replicating bacteria at 12 h postinfection are GFP positive and devoid of the proliferation dye. Extracellular bacteria and the macrophage plasmalemma are marked with tetramethyl rhodamine isothiocyanate (TRITC)-conjugated wheat germ agglutinin. These images are representative of several independent infections. In panel C, the fluorescent micrographs depict GFP-expressing bacteria that were employed in fluorescence-based proliferation assays. Shown is wild-type S. aureus USA300 carrying empty pALC2073 and the Δ*graS*(pALC2073) and Δ*graS*(pGraS) strains. Replicating bacteria are detected as green yet proliferation dye-negative bacteria at 12 h postinfection. White arrows point to proliferating bacteria, whereas arrowheads point to bacteria that have not replicated and are GFP and proliferation dye positive. Endogenous LAMP-1 protein detected by immunostaining at 12 h postinfection is shown in red. Bars equal 10 µm. These images are representative of three independent experiments. In panel D, the fraction of macrophages containing replicating bacteria normalized to the wild-type USA300 infection is shown. These data are the mean ± SEM from three independent experiments. Significance was determined by one-way ANOVA with Bonferroni’s posttest. *, *P* < 0.05; **, *P* < 0.001. In panel E, quantitation of the fraction of primary human M-CSF-derived macrophages containing replicating *graS* mutant bacteria normalized to the wild type is shown. The data are the mean ± SEM from three independent experiments in which macrophages were derived from three independent blood donors. Replicating bacteria appeared GFP positive but were negative for the far-red proliferation dye eFluor-670 at 12 h postinfection. Statistical significance was determined by an unpaired *t* test, and the asterisks indicate a *P* value of <0.0001. In panel F, eFluor-labeled GFP-expressing wild-type S. aureus USA300 and the *graS* strain are shown in primary human M-CSF-derived macrophages that were immunostained with anti-LAMP-1 antibody at 12 h postinfection. White arrows point to LAMP-1-positive bacteria. Bars equal ~10 µm.

In both the *in vitro* and *in vivo* settings, the growth of S. aureus in macrophage phagolysosomes is delayed, occurring more than 8 h postinfection (15, 16). Implicit in this observation is that S. aureus undergoes adaptation to the phagolysosomal environment, yet how this is accomplished is unknown. There is evidence to suggest that under certain conditions, the survival of S. aureus at acidic pH requires the GraXRS regulatory system (26). A hallmark of mature phagolysosomes is their marked acidity and, therefore, we hypothesized that S. aureus, as opposed to perforating the SaCP with toxins as we have just described does not occur, may simply adapt to this acidic environment through sensing by the GraXRS regulatory system. A mutant of S. aureus USA300 lacking the sensor kinase GraS gene (*graS*) was created, and its ability to survive and proliferate within RAW macrophages was assessed. Analysis of the fold change in CFU per milliliter at 12 h postinfection compared to 1.5 h postinfection revealed that wild-type bacteria increased on average 12.5 ± 3.9-fold (Fig. 3A). In contrast, the *graS* strain yielded a fold change in CFU per milliliter of 0.95 ± 0.29-fold during the same time frame, indicating the bacteria failed to grow (Fig. 3A). To visualize the *graS* mutant and confirm the inability of this strain to replicate within RAW macrophages, we also performed fluorescence-based proliferation assays. RAW macrophages were infected with wild-type USA300 or *graS* bacteria expressing GFP that were colabeled with the far-red proliferation dye, and after gentamicin treatment, infected macrophages were visualized at 1.5 and 12 h postinfection. At the early time point, neither strain had commenced replicating intracellularly (data not shown); however, at 12 h postinfection, macrophages harboring wild-type bacteria contained GFP-positive foci that were eFluor and wheat germ agglutinin (WGA) negative, indicating intracellularly replicating S. aureus cells were present (Fig. 3B, top panels). In contrast, the overwhelming majority of phagocytosed *graS* bacteria remained GFP and eFluor positive, indicating this strain has a replication-defective phenotype in macrophages (Fig. 3B, bottom panels), despite there being no detectable difference in their infectivities (see Fig. S4A in the supplemental material). Complementation experiments were performed to confirm the importance of *graS* for intracellular growth. In these experiments, *graS* bacteria carrying a vector control failed to grow within macrophages by 12 h postinfection, whereas wild-type and *graS* bacteria with *graS* expressed from a plasmid grew within RAW macrophages (Fig. 3C and D). Moreover, analysis of LAMP-1 protein confirmed again that wild-type and complemented bacteria replicate within LAMP-1-positive vacuoles (Fig. 3C). Similarly, the *graS* mutant is constrained by LAMP-1-positive membranes; however, these bacteria remain proliferation dye positive (Fig. 3C). Importantly, these data were recapitulated using primary human M-CSF-derived macrophages, where, once again, fluorescence proliferation assays confirmed that *graS*-deficient bacteria fail to replicate by 12 h postinfection (Fig. 3E and F). In summary, these data reveal that S. aureus does not alter the pH of the SaCP, instead requiring the sensor kinase GraS to adapt to this compartment prior to the initiation of bacterial replication.

10.1128/mBio.01143-18.5 FIG S4  Growth of S. aureus USA300 and *graS* bacteria at acidic or neutral pH. In panel A are shown the CFU per milliliter for WT USA300 and *graS* bacteria recovered from within RAW and BMDM macrophages at 1.5 h postinfection, indicating that there is no difference in infectivities between the two strains of bacteria. The data are the mean CFU per milliliter ± SEM from at least three independent experiments. n.s. denotes no statistical significant differences. In panel B, the growth of WT S. aureus USA300 and *graS* bacteria in 250 µl TSB buffered to pH 7.4 and 5.5 was monitored over time in a BioScreen automated growth curve analyzer. The graph depicts the mean ± SEM optical density reading (OD_600_) at each time point, and the data are derived from each strain cultured in biological triplicate for each condition. Download FIG S4, TIF file, 0.3 MB. Copyright © 2018 Flannagan et al. 2018 Flannagan et al. This content is distributed under the terms of the Creative Commons Attribution 4.0 International license.

### Phagolysosome acidification is a requirement for intracellular growth of S. aureus.

**FIG 4:**
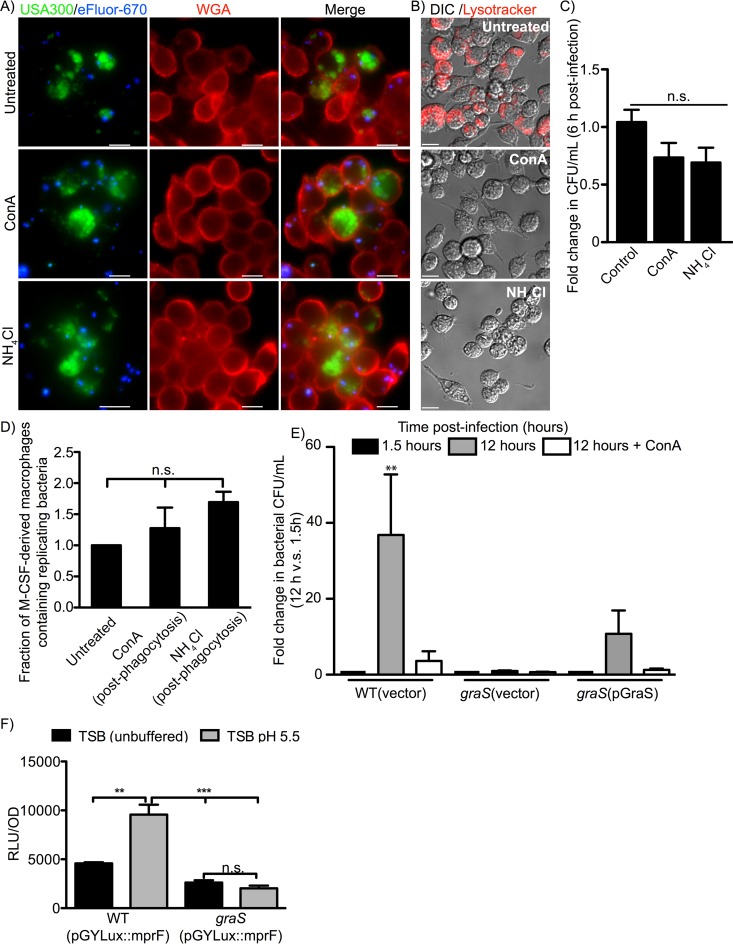
V-ATPase inhibition prior phagocytosis impairs S. aureus growth in macrophages, yet alkalinization of S. aureus-containing phagolysosomes is without effect. The effect of making phagolysosomes harboring live GFP-expressing and eFluor-labeled S. aureus USA300 more alkaline was examined. In panel A, fluorescence-based proliferation assays were performed, and the representative images depict growth of intracellular S. aureus at 12 h postinfection. Macrophages after infection and gentamicin treatment (i.e., at 1.5 h postinfection) were left untreated or were treated with 1 µM ConA or 40 mM NH_4_Cl, which were maintained on the cells throughout the experiment. These images are representative of at least three independent experiments. Scale bars equal ~10 µm. In panel B, the effect of ConA and NH_4_Cl on the ability of macrophages to maintain acidified endosomes and lysosomes as revealed by LysoTracker Red DND-99 staining is shown. Images are of living cells stained with LysoTracker after pretreatment with either 1 µM ConA, 40 mM NH_4_Cl, or no treatment at all and are representative of two independent experiments. Scale bars equal ~10 µm. In panel C, the bacterial burden of RAW macrophages in the presence or absence of ConA and NH_4_Cl is shown. Data are the mean ± SEM fold change in CFU per milliliter at 6 h postinfection and are derived from four independent experiments. n.s. indicates not significant as determined by one-way ANOVA with a Dunnett’s posttest *P* value of >0.05. In panel D, the effect of ConA and NH_4_Cl on phagolysosomal replication of GFP-expressing S. aureus in primary human M-CSF-derived macrophages is shown. Fluorescence-based proliferation assays were performed as in panel A. The graph shows the fraction of macrophages that contained replicating bacteria in the presence or absence of ConA and NH_4_Cl at 12 h postinfection. The data are the mean ± SEM derived from three independent experiments using macrophages derived from three independent blood donors. n.s. denotes not significant with *P* > 0.05. The data presented in the graph have been normalized to the untreated condition for each experiment. In panel E, the bacterial burden of RAW macrophages infected with USA300 and the *graS* mutant carrying either the vector control or the GraS complementation vector is shown. Macrophages were also pretreated with 1 µM ConA prior to infection to inhibit V-ATPase function and lysosome acidification prior to the uptake of S. aureus. The data presented are the mean fold change in CFU per milliliter ± SEM at the indicated time relative to the bacterial burden obtained of each respective strain at 1.5 h postinfection (after gentamicin treatment) in the presence and absence of ConA. These data were derived from at least three experiments, and significance was determined by one-way ANOVA with Bonferroni’s posttest. **, *P* ≤ 0.01. In panel F, the graph shows the relative light production from wild-type S. aureus USA300 and the *graS* mutant each carrying plasmid pGYLux::*mprF*. The plotted data are the mean RLU ± SEM after 2 h of growth from a representative experiment in which each strain was analyzed in biological triplicate for each condition. These data were background subtracted, where background was taken as each strain transformed with empty pGYLux plasmid and cultured under the same conditions. Statistical significance was determined by one-way ANOVA with a Bonferroni’s posttest. **, *P* < 0.01; ***, *P* < 0.0001.

Given our assertion that S. aureus must first sense acidic pH to be able to adapt to the phagolysosome prior to the commencement of replication, it would be reasonable to posit that alkalinization of the phagolysosome would accelerate growth of wild-type S. aureus. To test this, we performed fluorescence-based proliferation assays in which phagocytes were treated with ConA or the weak base NH_4_Cl after the bacterial trafficking to the phagolysosome (i.e., at 1.5 h postinfection). This analysis revealed that at 12 h postinfection, S. aureus had replicated intracellularly; however, inhibitor treatment did not enhance growth compared to that of control macrophages (Fig. 4A). Using the acidotropic dye LysoTracker Red, we could verify that our ConA and NH_4_Cl treatment regimen had the intended affect and alkalinized lysosomes as expected (Fig. 4B). In parallel, we performed gentamicin protection assays, which also revealed that ConA or NH_4_Cl treatment did not accelerate intracellular growth of S. aureus compared to control RAW macrophages (Fig. 4C). Finally, these results were recapitulated in primary human M-CSF-derived macrophages, where fluorescence proliferation assays revealed that ConA and NH_4_Cl treatment did not augment S. aureus growth (Fig. 4D).

While alkalinization of phagolysosomes alone did not improve bacterial replication, we next considered whether V-ATPase inhibition could protect *graS* bacteria and improve their growth in macrophages. To this end, we infected RAW macrophages with wild-type S. aureus USA300 carrying pALC2073 (empty vector), the S. aureus *graS* strain carrying pALC2073, and the S. aureus *graS* strain carrying the complementation plasmid pGraS, and performed gentamicin protection assays to assess changes in bacterial burden inside macrophages over time. To protect *graS* bacteria from any exposure to acid stress, we now pretreated macrophages with ConA, which was maintained throughout the infection. In these infections, the bacterial burden for wild-type S. aureus USA300(pALC2073) increased ~38-fold by 12 h postinfection, whereas pretreatment of macrophages with ConA surprisingly impaired wild-type growth (Fig. 4E). Consistent with our earlier observations (Fig. 3), we found that *graS*-deficient S. aureus did not proliferate within macrophages by 12 h postinfection, and pretreatment of macrophages with ConA failed to rescue bacterial growth (Fig. 4E). Importantly, complementation with *graS* in *trans* restored growth, as evidenced by the ~13-fold increase in bacterial burden in untreated macrophages. Interestingly, pretreatment of macrophages with ConA also impaired proliferation, as was observed for wild-type S. aureus (Fig. 4E). Taken together, these data indicate that exposure to acid in the phagolysosome engenders S. aureus with the ability to grow in this niche, and this requires the sensor kinase GraS. Consistent with this, we find that, when grown *in vitro* at pH 5.5 to mimic the measured pH of the phagolysosome, *graS*-deficient S. aureus cells grow similarly to wild-type bacteria, indicating that this level of acidity alone cannot account for the observed growth defect inside the SaCP (Fig. S4B). Finally, to determine whether the GraS sensor can perceive pH alone, we constructed a bioluminescent reporter to monitor the promoter activity of *mprF*, an established GraS-regulated gene. In wild-type S. aureus strain USA300, plasmid pGYLux::*mprF* made the bacteria bioluminescent, which was enhanced upon culture at pH 5.5 (Fig. 4F). In contrast, induction of bioluminescence at pH 5.5 was abrogated in *graS* bacteria carrying the same pGYLux::*mprF* plasmid (Fig. 4F), indicating *mprF* promoter activity is enhanced at acidic pH, via GraS.

### GraS is required for acidic pH-dependent resistance of S. aureus to killing by polymyxin B and is required for resistance to LL-37 and reactive oxygen species.

**FIG 5:**
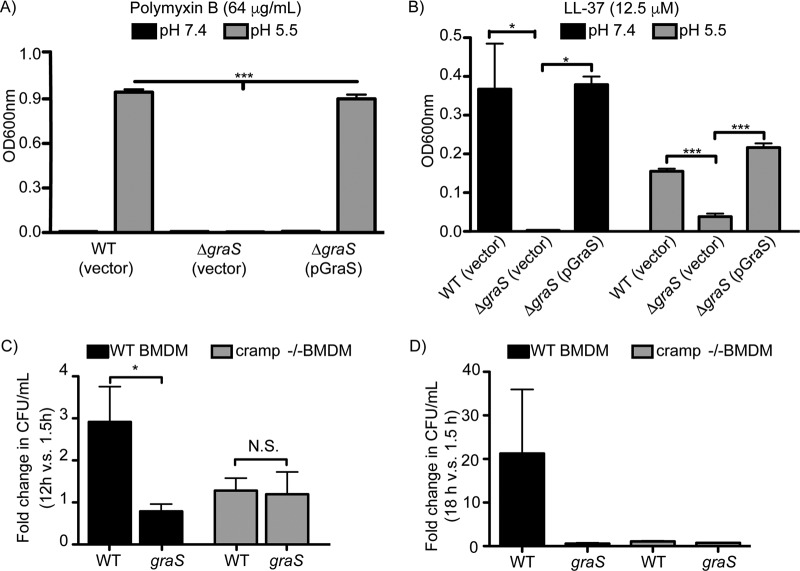
Acidic pH induces S. aureus resistance to select antimicrobial peptides, and peptide resistance requires *graS*. The resistance of S. aureus USA300 and a mutant strain lacking the *graS* gene to PmB (A) and LL-37 (B) in response to changes in pH is shown. In panel A, S. aureus USA300 carrying the vector control (pALC3073) and a *graS* mutant carrying either a vector control (pALC2073) or a GraS-encoding plasmid (pGraS) were cultured in RPMI 1640 at pH 7.4 or 5.5 in the presence 64 µg/ml polymyxin B (PmB). Shown is the endpoint OD_600_ after 24 h of growth under such culture conditions. The data are the mean OD_600_ ± SEM from three independent experiments with each strain cultured in biological triplicate for each. In panel B, similar experiments were performed, except the bacteria were cultured in the presence of 12.5 µM human LL-37 (B). In panels A and B, statistical significance was determined by Student’s unpaired *t* test. *, *P* ≤ 0.05; ***, *P* ≤ 0.001. In panels C and D, the ability of S. aureus USA300 and *graS*-deficient bacteria to proliferate in wild-type and *cramp*^−/−^ bone marrow-derived macrophages after infection at an MOI of 10 is shown. In panel C, the fold change in bacterial burden at 12 h versus 1.5 h postinfection is shown. These data are the mean ± SEM from three independent experiments with each strain analyzed in biological triplicate. *, *P* < 0.05 as determined by an unpaired Student’s *t* test; N.S., not statistically significant. In panel D, the fold change in bacterial burden at 18 h postinfection, normalized to 1.5 h postinfection, is shown. These data are the mean ± SEM from a single experiment with each strain analyzed in 3 to 5 independent replicates.

Previous work has shown that the GraXRS regulatory system is required for antimicrobial peptide (AP) resistance and that AP resistance can be modulated by acidic pH (26, 27). Presumably in the phagolysosome of the macrophage, these factors and others are experienced by S. aureus simultaneously and GraS plays an important role in enabling bacterial adaptation. Here, we explored the role GraS plays in mediating AP resistance at pH 5.5 to mimic the measured pH of the macrophage phagolysosome (Fig. 5A and B). To this end, the S. aureus USA300(pALC2073), *graS*(pALC2073), and *graS*(pGraS) strains were cultured in serum-free RPMI at pH 7.4 and 5.5 in the presence or absence of 64 µg/ml polymyxin B (PmB) or 12.5 µM human LL-37. All three strains, although able to grow in RPMI alone, failed to grow in RPMI in the presence of PmB (Fig. 5A). In contrast, at pH 5.5 and in the presence of PmB, only the wild-type S. aureus USA300(pALC2073) and *graS*(pGraS) strains grew well in the presence of PmB (Fig. 5A). Cultures of S. aureus in the presence of the human cathelicidin LL-37 at neutral and acidic pH did not demonstrate such dramatic differences in LL-37 resistance at pH 7.4 and 5.5 (Fig. 5B). Nevertheless, growth of the *graS*(pALC2073) strain was significantly reduced relative to that of wild-type and *graS*(pGraS) bacteria in the presence of LL-37 at pH 7.4 and 5.5, indicating GraS is required for LL-37 resistance under both conditions (Fig. 5B).

Within the phagolysosome, S. aureus would be expected to experience reactive oxygen species (ROS)-imposed stress, and previous work has indicated that GraXRS contributes to ROS resistance (28). To determine whether ROS resistance in S. aureus is influenced by GraS and pH, the same strains were cultured in RPMI at pH 7.6 and 5.7 in the presence and absence of 10 µM paraquat (see Fig. S5 in the supplemental material). Remarkably, in the presence of paraquat at pH 7.6, all strains demonstrated very poor growth, but this growth impairment was alleviated at acidic pH (Fig. S5). Under these conditions, wild-type S. aureus USA300 grew on average ~32% better than the *graS* strain; however, in this instance, providing GraS in *trans* failed to complement the growth difference (Fig. S5). Taken together, these data indicate that GraS is required for S. aureus resistance to antimicrobial effectors found in phagolysosomes and bacterial killing can be affected by environmental pH.

10.1128/mBio.01143-18.6 FIG S5  pH-dependent induction of resistance to reactive oxygen species stress. The ability of S. aureus USA300 and a mutant strain lacking the *graS* gene (strain H3544) to grow in the presence of 10 µM paraquat at pH 7.6 (A) and 5.7 (B) was analyzed. In panels A and B, S. aureus USA300 carrying the vector control (pALC3073) and a *graS* mutant carrying either a vector control (pALC2073) or a GraS-encoding plasmid (pGraS) were cultured in RPMI 1640 at the indicated pH with and without paraquat. The graph depicts the endpoint OD_600_ reading for each strain after 24 h of growth at 37°C. The data are the mean ± SEM from three independent experiments with each strain cultured as biological duplicate samples. Significance was determined by one-way ANOVA with Bonferroni’s posttest. **, *P* < 0.01; ***, *P* < 0.0001. Download FIG S5, TIF file, 0.3 MB. Copyright © 2018 Flannagan et al. 2018 Flannagan et al. This content is distributed under the terms of the Creative Commons Attribution 4.0 International license.

### GraS-deficient and wild-type S. aureus USA300 strains fail to proliferate in *cramp*^−/−^ macrophages.

Given that inactivation of *graS* results in sensitivity of S. aureus to antimicrobial peptides, we sought to determine whether infection of cathelicidin-deficient murine macrophages could rescue the growth defect of *graS* bacteria. To this end, macrophages were derived, using M-CSF, from the bone marrow of wild-type C57BL/6 and *cramp*^−/−^ mice. The bone marrow-derived macrophages (BMDMs) were infected with either S. aureus USA300 or a *graS* mutant, and at 1.5 h postinfection, similar numbers of wild-type and mutant bacteria were recovered from macrophages of both backgrounds. By 12 h postinfection in C57BL/6 BMDMs, wild-type S. aureus USA300 had increased approximately 3-fold, whereas *graS* bacteria failed to proliferate (Fig. 5C). In *cramp*^−/−^ M-CSF-derived macrophages, *graS* bacteria also failed to proliferate by 12 h postinfection; however, remarkably, USA300 also repeatedly failed to proliferate (Fig. 5C). Due to this striking observation, subsequent macrophage infections were carried out until 18 h postinfection to determine whether bacterial growth was simply delayed in *cramp*^−/−^ macrophages (Fig. 5D). By 18 h postinfection, wild-type S. aureus USA300, in the presence of wild-type macrophages, grew to a high cell density due to the eventual escape from infected phagocytes and extracellular growth, as has been previously described (15) (Fig. 5D). In contrast, *graS* bacteria remain unable to proliferate even after 18 h in both wild-type and *cramp*^−/−^ macrophages. In stark contrast, even at 18 h postinfection wild-type S. aureus USA300 failed to commence replicating in *cramp*^−/−^ macrophages (Fig. 5D). These data suggest that S. aureus uses the presence of cathelicidin in the phagolysosome as a signal, through the Gra two-component regulatory system, to adapt to this hostile environment before proliferation proceeds.

### Methicillin-sensitive S. aureus strain Newman requires GraS to grow within macrophages.

**FIG 6:**
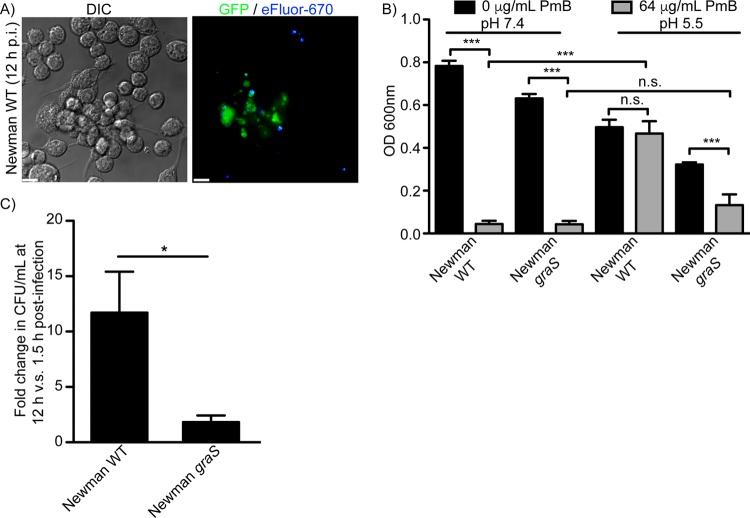
Growth of methicillin-sensitive S. aureus strain Newman in the presence of PmB and in macrophages requires *graS*. In panel A, the micrographs depict S. aureus strain Newman proliferating at 12 h postinfection in RAW macrophages. At this time, GFP-positive yet proliferation dye-negative bacteria represent S. aureus that had undergone intracellular replication. Scale bars equal 10 µm. In panel B, the ability of S. aureus strain Newman and a derivative lacking a functional *graS* gene to grow in the presence of PmB is shown. Wild-type and *graS* bacteria were cultured at pH 7.4 and 5.5 in the presence and absence of 64 µg/ml PmB, and the optical density (OD_600_) was measured after 20 h of growth. The data are the mean OD_600_ reading ± SEM with each strain analyzed in biological triplicate in three independent experiments. ***, *P* ≤ 0.001. In panel B, the inability of the *graS* mutant in the Newman background to grow inside macrophages is shown. Growth of the parental strain and the mutant expressed as a mean fold change ± SEM in CFU per milliliter at 12 h versus 1.5 h postinfection is shown. Statistical significance was determined by a Student’s unpaired *t* test. *, *P* ≤ 0.05.

Inactivation of GraS in S. aureus USA300 significantly impairs the ability of these bacteria to grow within the macrophage phagolysosome; however, we sought to confirm that this was not specific to the USA300 strain. We first demonstrated that the methicillin-sensitive S. aureus strain Newman can replicate inside RAW cells (Fig. 6A). Therefore, we transduced the *graS*::φNΣ mutation from the Nebraska transposon library into strain Newman. As a first step toward characterizing this strain, we sought to confirm that inactivation of *graS* renders Newman sensitive to antimicrobial peptide-dependent killing. To this end, we compared the growth of wild-type Newman and its isogenic *graS* mutant in the presence and absence of 64 µg/ml PmB at pH 7.4 and 5.5 (Fig. 6B). Consistent with our USA300 data, we find that at neutral pH, neither wild-type nor mutant bacteria grow in the presence of 64 µg/ml PmB (Fig. 6B). In contrast at pH 5.5, strain Newman deficient for *graS* is highly sensitive to PmB and grows significantly less than the wild-type or the *graS* mutant in the absence of PmB. In contrast, wild-type Newman bacteria grow equally well in the presence and absence of PmB at pH 5.5, indicating that indeed in the methicillin-sensitive S. aureus background, *graS* is required for PmB resistance even at acidic pH (Fig. 6B). Next, we analyzed the ability of the Newman *graS* strain to replicate inside RAW macrophages. By 12 h postinfection, wild-type Newman achieves on average an ~10-fold increase in bacterial burden, which contrasts with the *graS* strain, which failed to replicate (Fig. 6C). Taken together, these data indicate that GraS is important for the intracellular replication of S. aureus and is not a unique requirement in strain USA300.

### GraS-regulated *mprF* is required for S. aureus replication in macrophages.

**FIG 7:**
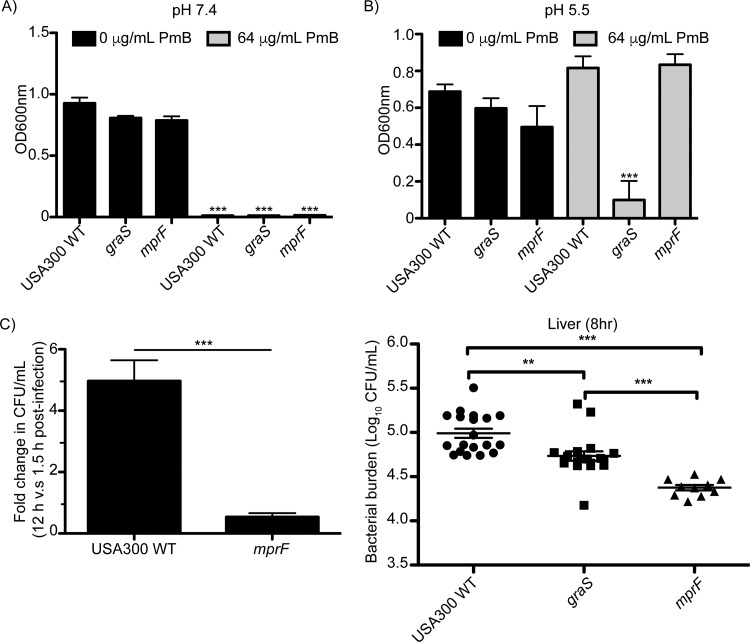
GraS and the downstream gene *mprF* are required for growth in macrophages and optimal survival in the murine liver in the early stages of systemic infection. The ability of S. aureus lacking the *mprF* gene to resist PmB-dependent growth restriction at pH 7.4 (A) and 5.5 (B) was determined. In panels A and B, bacteria were cultured in RPMI at the indicated pH in the presence and absence of 64 µg/ml PmB and growth was measured after 20 h at 37°C. Shown are the mean ± SEM optical density readings from two independent experiments with each strain cultured in biological triplicate in each experiment. In panel C, the ability of *mprF*-deficient S. aureus to grow inside RAW macrophages is shown. The data are the mean ± SEM from three independent experiments and are expressed as the fold change in CFU per milliliter at 12 h postinfection for wild-type S. aureus USA300 and the *mprF* mutant. The data were derived from at least three independent experiments, and statistical significance was determined by a Student’s unpaired *t* test (*P* ≤ 0.0001). The role of *graS* and *mprF* in the survival of S. aureus in the murine liver was determined at 8 h postinfection, when the bacteria are expected to reside within Kupffer cells. Mice infected with S. aureus via tail vein injection were sacrificed, and the bacterial burden in whole-liver homogenates was determined. The data are presented as the log_10_ CFU per milliliter, and each symbol represents the bacterial burden from one infected animal. The horizontal bars represent the mean ± SEM. Statistical significance was determined by a one-way ANOVA with Bonferroni’s posttest to compare each mean. **, *P* ≤ 0.01; ***, *P* ≤ 0.001.

The multiple peptide resistance factor MprF catalyzes the formation of lysylphosphatidylglycerol (LPG) and contributes to antimicrobial peptide resistance in S. aureus. Moreover, as we have shown above, *mprF* promoter is activated in response to acidic pH through GraS (Fig. 4F). As such, we speculated that MprF might contribute to the survival of S. aureus within the phagolysosome. To analyze the importance of *mprF* for the replication of S. aureus in the macrophage, we utilized a mutant from the Nebraska transposon library carrying an inactivated copy of the *mprF* gene (*mprF*::φNΣ). As a first step toward characterizing the *mprF* mutant, growth of wild-type S. aureus USA300, USA300 *graS*, and USA300 *mprF* in the presence and absence of 64 µg/ml PmB at pH 7.4 and 5.5 was analyzed (Fig. 7A). As expected, at neutral pH each strain failed to grow in the presence of PmB yet grew equally well in the absence of the peptide (Fig. 7A). In contrast, at pH 5.5 wild-type S. aureus USA300 and the *mprF* mutant grew to similar optical densities in the presence of PmB, while the *graS* strain was dramatically impaired for growth (Fig. 7B). While these data indicated that the gene(s) downstream of GraS that contribute to PmB resistance must not be *mprF*, at least on its own, we considered that MprF might still play an important role for growth in the phagolysosome. To analyze this, we performed gentamicin protection assays using RAW macrophages in which the abilities of S. aureus USA300 and the *mprF* strain to grow over a 12-h infection were compared. In these experiments, wild-type S. aureus USA300 strain demonstrated an ~5-fold increase in bacterial density at 12 h compared to 1.5 h postinfection (Fig. 7C). In contrast, the *mprF* mutant when analyzed in parallel failed to yield an increased bacterial burden (Fig. 7C). These data indicate that *mprF* contributes to phagolysosomal growth of S. aureus but not to resistance to PmB at pH 5.5.

### GraS and MprF are required for early stage survival of S. aureus within the murine liver.

As our data show *graS* is required for the ability of S. aureus to proliferate within the macrophage phagolysosome *in vitro*. Therefore, we next investigated whether GraS would have a role in bacterial survival *in vivo* in the acute or early stages of a systemic infection. It has been demonstrated that during systemic infection, S. aureus is readily captured by Kupffer cells, the resident macrophages in the liver, and once captured reside in these cells for hours before they then begin to replicate (16). Since S. aureus remains confined to Kupffer cells at 8 h postinfection, we employed this model to determine whether GraS would contribute to bacterial survival in macrophages *in vivo*. To this end, BALB/c mice were injected via tail vein with ~5.0 × 10^6^ cells of wild-type S. aureus USA300 or the *graS* or *mprF* mutant strain, and at 8 h postinfection, the bacterial burden for each strain in the murine liver was determined. At this time, each strain could be recovered from the liver, indicating none of the strains was completely eradicated. However, we found that significantly more wild-type S. aureus USA300 bacteria can be recovered from the liver 8 hpi compared to mice infected with the *graS* or *mprF* bacteria. These data indicate that GraS and the GraS-regulated MprF contribute to S. aureus survival in the liver, early in infection (i.e., 8 hpi), and presumably in Kupffer cells (Fig. 7D).

## DISCUSSION

Staphylococcus aureus is a versatile pathogen seemingly capable of infecting and surviving in any niche of the body, illustrating the remarkable ability of this bacterium to circumvent immune mechanisms of the host. At the cellular level, the macrophage phagolysosome represents a highly microbicidal environment, and yet S. aureus proliferates within this niche (15, 16). How S. aureus evades killing and replicates inside phagolysosomes has not been determined, and here we demonstrate that S. aureus requires the sensor kinase GraS for phagolysosomal growth in murine and human macrophages. Moreover, we show that GraS contributes to S. aureus survival *in vivo* in the acute stages of systemic infection, coinciding with when the bacteria have been shown to reside in Kupffer cells (16). As an antimicrobial organelle, the phagolysosome is acidic, it can be enriched with toxic ROS, and it contains antimicrobial proteins and peptides (i.e., lysozyme and cathelicidin) that intoxicate ingested bacteria (reviewed in reference 29). In S. aureus, GraXRS regulates genes (e.g., *mprF*, *dltABCD*, and *vraFG*) (28, 30, 31) involved in antimicrobial peptide resistance, and our observation that bacteria lacking GraS are unable to replicate within the macrophage phagolysosome further supports this notion. Here we have shown through fluorescence imaging that, in the context of the macrophage, S. aureus resides inside intact LAMP-1-positive phagolysosomes that are fully acidified, and this is where proliferation of phagocytosed S. aureus commences. Moreover, it is within these LAMP-1-positive phagolysosomes where *graS* cocci, despite being hemolytic (Fig. S1A), remain confined at least within the time frame (through 12 h postinfection) considered here.

Unlike other successful intracellular pathogens (e.g., Listeria monocytogenes and Mycobacterium tuberculosis) that disrupt phagosome maturation to evade phagolysosome fusion (reviewed in reference 11), we find that S. aureus resides inside mature phagolysosomes. Consequently, we find that acidic pH, a hallmark of phagolysosome fusion, acts as an environmental cue that elicits adaptation and increases the ability of S. aureus to replicate within this niche. We find that GraXRS is required for growth inside the macrophage phagolysosome, and through GraS, we find that S. aureus upregulates, in a pH-dependent manner, the expression of genes (e.g., *mprF*) that will promote survival in this antimicrobial compartment. Consistent with our observations, recent work has indicated that GraXRS is used by S. aureus to adapt and grow at acidic pH *in vitro*, independently demonstrating the GraS sensor allows S. aureus to perceive changes in environmental pH (32). The GraXRS system is known to play an important role in regulating antimicrobial peptide resistance in S. aureus, and our data further support this assertion. Here we find that acidic pH can modulate the intrinsic resistance of S. aureus to the antimicrobial compound PmB; however, the same effect is not apparent for LL-37, at least under the experimental conditions analyzed here, indicating the mechanisms of resistance for these two peptides are likely different. This is exemplified by inactivation of the GraS-regulated gene *mprF*, which has been shown to confer resistance to defensins (33), but does not render S. aureus sensitive to PmB even at acidic pH (Fig. 7B). Nevertheless, in the acidic milieu of the phagolysosome, S. aureus would upregulate Gra-regulated genes that through different mechanisms should allow the bacteria to better resist a broad-spectrum antimicrobial attack. Previous work has indicated that GraS in S. aureus is responsive to exposure to antimicrobial peptides as well (28, 31), and conceivably, APs and acidic pH are “sensed” by GraS. Detailed studies of the GraS sensor have revealed that its periplasmic loop and several critical residues contained therein are required for GraS signal transduction in response to antimicrobial peptide exposure (26, 34). It will be of interest to determine if the same residues in GraS are required for its pH-responsive functions as well as it may very well be a combination of these stimuli that leads to adaptation and growth of S. aureus in the macrophage phagolysosome. In agreement with this notion, we find that even in *cramp*^−/−^ macrophages, wild-type S. aureus shows impaired growth compared to infection in wild-type macrophages. Conceivably, this is due to the absence of cathelicidin, which could operate as an environmental cue to which S. aureus adapts. Consistent with the importance of such cues for S. aureus growth, we also find that alkalinization of macrophage lysosomes, by pretreatment with an inhibitor of the V-ATPase, also impairs the intracellular growth of wild-type S. aureus USA300 in wild-type macrophages. This is in agreement with previous observations (18, 35), and the inability of S. aureus to grow in the absence of cathelicidin or phagosome acidification may not be unrelated. Indeed, cathelicidin is synthesized as a preprotein that must be proteolytically processed to become an active antimicrobial peptide (36). Given that lysosomal alkalinization impairs proteolytic activity (37), it is tempting to speculate that V-ATPase inhibition also abrogates cathelicidin processing. In this way, two stimuli that have now been shown to evoke GraS signaling would be eliminated. At the outset of these experiments, it was hypothesized that inhibition of phagosome acidification or the absence of cathelicidin would augment S. aureus growth, as has been observed for other bacterial pathogens (38, 39). However, from our experiments, it is clear that the interaction of S. aureus with the phagolysosomal environment is complex and additional antimicrobial effectors must also restrict S. aureus growth.

Previous work has suggested that S. aureus deploys toxins such as Hla or PSMα peptides to mediate escape from the S. aureus-containing phagosome or to allow the bacteria to replicate within the macrophage (19, 20). The notion that S. aureus must escape the phagosome to replicate is incompatible with our molecular imaging data showing the subcellular niche in which phagocytosed S. aureus replicates is also acidic. Indeed, perforation of the limiting SaCP would lead to dissipation of any proton gradient and alkalinization of the vacuole. Moreover, the observation that *graS* bacteria, which are hemolytic, are unable to replicate within the macrophage challenges the notion that toxin production is required for S. aureus growth inside the macrophage. Many toxins (e.g., Hla, PSMα peptides, and LukAB) are either directly or indirectly regulated by Sae and Agr (21, 40), and considering the receptors (e.g., ADAM-10 and CD11b) required for cytotoxicity are displayed at the cell surface (41, 42), it is perhaps not unexpected that these systems are dispensable for growth inside the macrophage phagosome. It is important to emphasize that, in our study, we have focused on the ability of intracellular bacteria to grow inside the phagolysosome in the absence of an extracellular infection; in the latter case, it is well known that Agr- and Sae-regulated toxin production plays an important role in leukocyte intoxication (43–46). As mentioned above, Agr and Sae have been implicated in the ability of S. aureus to withstand the innate defenses of neutrophils and macrophages (18, 22, 24); however, our data reveal that these systems are dispensable for the replication of S. aureus in the macrophage phagolysosome. Presumably, the discrepancy between previous studies and the data we present here, at least in terms of the role that Agr plays in intracellular survival of S. aureus, is due to strain variation, use of distinct cells and or cell lines, and methodological differences, namely, the prolonged use of antibiotics. The notion that aminoglycoside antibiotics such as gentamicin, which are commonly used for *in vitro* infection assays, do not enter host cells is not accurate and is beginning to recede (15, 47–49). Nevertheless, several studies to date investigating macrophage infection by S. aureus have employed prolonged antibiotic treatment, which will have undoubtedly led, in some instances, to the characterization of intracellular antibiotic-treated bacteria. Regardless, the realization that *graS* is required for S. aureus proliferation within the macrophage phagolysosome and that acidic pH plays an important role in eliciting a GraS response represents an important step toward understanding, at the molecular level, how S. aureus can circumvent the innate immune function of macrophages in the intracellular environment.

## MATERIALS AND METHODS

### Reagents.

The fluorescent cell proliferation dye eFluor-670 was from eBiosciences. Tetramethyl rhodamine isothiocyanate (TRITC)-conjugated wheat germ agglutinin (TMR-WGA), LysoTracker green DND-26, and FITC-dextran (molecular weight [MW], 10,000) were from Thermo Fisher Scientific. Concanamycin A (ConA) was purchased from Santa Cruz Biotechnology. Goat anti-human Alexa 488-, goat anti-human Cy3-, and goat anti-rat Cy3-conjugated antibodies were from Jackson ImmunoResearch, Inc. All restriction enzymes and T4 DNA ligase were from New England Biolabs, Inc. Polymyxin B, paraquat dichloride, and NH_4_Cl were purchased from Sigma-Aldrich. LL-37 was purchased from AnaSpec, Inc.

### Bacterial strains, bacterial plasmids, and culture conditions.

Escherichia coli DH5α was used for cloning purposes and was cultured as previously described (50). All S. aureus strains and the plasmids used in this study are summarized in Table 1. Routine culture of all S. aureus was done as previously described (15) with antibiotics at the following concentrations as needed: erythromycin, 3 µg/ml; chloramphenicol, 12 µg/ml; lincomycin, 20 µg/ml; and tetracycline, 4 µg/ml.

For growth of S. aureus in tryptic soy broth (TSB) buffered at pH 7.4 and 5.5, TSB Tris-maleate buffer (0.1 M Tris and 0.1 M maleic acid) was used in place of water. pH stability of sterile medium was monitored prior to each experiment, and maintenance of pH in spent culture medium was monitored after each experiment. For growth curves at pH 7.4 and 5.5, bacterial proliferation was monitored using a BioScreen automated growth curve analyzer with 250-µl culture volumes and constant shaking at 37°C.

### S. aureus mutant construction.

All gene deletions in S. aureus USA300 were performed using the procedures previously described (51). In some instances, phage 80α was used for transduction following standard procedures. Detailed methods used for the creation of each mutant strain are provided in Text S1 in the supplemental material.

10.1128/mBio.01143-18.1 TEXT S1  Details for the creation of S. aureus mutants and plasmid construction. Additional methods related to ratiometric pH measurements are also provided. Download TEXT S1, DOCX file, 0.1 MB. Copyright © 2018 Flannagan et al. 2018 Flannagan et al. This content is distributed under the terms of the Creative Commons Attribution 4.0 International license.

### Growth of S. aureus in the presence of polymyxin B, LL-37, or paraquat.

Cells were cultured overnight in TSB at 37°C with shaking in the presence of antibiotics as necessary, pelleted, washed 1× with sterile saline, and then diluted to make a suspension with an optical density at 600 nm (OD_600_) of 0.5. The cells were then diluted 1:100 into 1 ml bicarbonate-free RPMI 1640 set to pH 7.4 or 5.5 with and without antimicrobial peptide at the following final concentrations: PmB, 64 µg/ml; and LL-37, 12.5 µM. Paraquat was used at a final concentration of 10 micro molar and in RPMI at pH 7.4 and 5.7. Optical density was evaluated after incubation with shaking at 37°C for 20 h, at which time an endpoint OD_600_ reading was measured.

### Bioluminescence assay conditions.

Overnight cultures of USA300(pGylux::*mprF*), USA300 *graS*(pGylux::*mprF*), and USA300 with empty vector pGylux were grown to stationary phase in TSB with chloramphenicol. Three biological replicate cultures were inoculated to an OD_600_ of 0.01 in either 25 ml of unbuffered TSB or TSB with 0.1 M MES (morpholineethanesulfonic acid) at pH 5.5 in a 125-ml Erlenmeyer flask and were grown at 37°C with 220 rpm. After 2 h, the number of relative light units (RLU) for each flask was measured 4 times, and the corrected RLU was calculated as the mean of these measurements minus the RLU for each strain carrying empty vector pGYlux cultured under identical conditions.

### Mammalian tissue culture.

RAW 264.7 macrophages and primary human M-CSF-derived macrophages were cultured as described previously (15). Blood was drawn from healthy volunteers in accordance with protocols approved by the University of Western Ontario Research Ethics Board. For all infections, macrophages were seeded into 12-well tissue culture dishes with 18-mm sterile glass coverslips as necessary.

### Macrophage infection assays.

All macrophage infections were performed as previously described (15). In brief, S. aureus cells grown overnight in TSB that were unopsonized were used to infect macrophages at a multiplicity of infection (MOI) of 10 unless otherwise indicated. Bacteria were washed once and diluted into serum-free RPMI 1640 medium (SF-RPMI). For the synchronization of infection, tissue culture plates were centrifuged for 2 min at 277 × *g* and then incubated for 30 min at 37°C in a humidified incubator with 5% CO_2_. After 30 min, the medium was aspirated and replaced with SF-RPMI containing 100 µg/ml gentamicin for 1 h. After gentamicin treatment, infected cells were rinsed with 1 ml sterile 1× phosphate-buffered saline (PBS) and replaced with RPMI-containing serum. When necessary, infections were incubated with 1 µg/ml TMR-WGA for 2 min prior to 20 min of fixation in 4% (vol/vol) paraformaldehyde (PFA) at room temperature. Alternatively, infected macrophages were lysed in 0.5 ml PBS containing 0.1% (vol/vol) Triton X-100 and serially diluted for CFU determination. In some instances, ConA (1 µM) or NH_4_Cl (40 mM) was used to treat macrophages prior to or after phagocytosis as appropriate. Once added, ConA and NH_4_Cl were maintained throughout the duration of the experiment.

To enumerate gentamicin-protected bacteria and calculate the fold change in CFU per milliliter, macrophages were lysed in 0.5 ml 0.1% (vol/vol) Triton X-100 and serially diluted in sterile saline. The fold change in CFU per milliliter for each experiment was determined by dividing bacterial counts obtained at 6 or 12 h postinfection for a given strain by the count obtained for the same strain at 1.5 h postinfection (i.e., right after gentamicin treatment). This was done for every condition in every experiment.

### Isolation of BMDMs.

Wild-type C57BL/6 mice and B6.129X1-*Camp*^*tm1Rlg*^/J male mice that were age matched were ordered from The Jackson Laboratory. At 6 to 8 weeks of age, bone marrow cells were extracted and cultured using standard procedures (52, 53). In brief, isolated bone marrow cells were plated at a density of 1 × 10^6^ cells per 18-mm well of a 12-well tissue culture dish containing a sterile cover glass. Cells were differentiated and maintained in 1 ml of RPMI supplemented with 10% (vol/vol) non-heat-inactivated fetal bovine serum (FBS), 10 ng/ml recombinant murine M-CSF (PeproTech), and antibiotics. The medium was changed on days 3 and 5 postisolation; however, on day 5, cells were washed four times with sterile 1× PBS and maintained in the same medium but without antibiotic. Macrophages were utilized for phagocytosis assays on day 7 and were infected with S. aureus as described above.

### Fluorescence proliferation assays.

Proliferation assays were done as previously described (15), and macrophage infections were performed as described above. In some instances, dead bacteria were used for phagocytosis. To inactivate S. aureus, bacteria were first labeled with eFluor-670, fixed with 4% (vol/vol) PFA for 20 min at room temperature, and then incubated for 35 min at 57°C. Cells were then washed and diluted in SF-RPMI for infections as described above. The fluorescent spectra of GFP or mCherry are sufficiently different from that of eFluor-670, such that the emission of GFP and eFluor or mCherry and eFluor can easily be separated using conventional filter sets. Due to the fact that all of the bacteria at the start of an infection are positive for eFluor-670, of which there is a finite amount, the appearance of eFluor-negative bacteria within macrophages identifies bacterial populations that have replicated which appear negative due to dilution of the eFluor dye below detectable levels.

### LAMP-1 immunostain.

Detection of endogenous LAMP-1 in primary human M-CSF-derived macrophages and RAW 264.7 macrophages was done as previously described (15). Mouse anti-human LAMP-1 antibody (H4A3) and rat anti-mouse LAMP-1 antibody (1D4B) were both purchased as supernatants from the Developmental Studies Hybridoma Bank (DSHB), and the antibodies were deposited into the DSHB: H4A3 by J. T. August and J. E. K. Hildreth and 1D4B by J. T. August.

### Dextran pulse-chase.

RAW and primary human macrophages adhered to 18-mm glass coverslips were cultured overnight (~16 h) in RPMI 1640 with 5 or 10% FBS (vol/vol), respectively, and loaded with FITC-dextran (100 µg/ml) as previously described (29). Infections were performed as described above, and at 11 h postinfection, infected macrophages were analyzed by live-cell fluorescence imaging.

### Assessment of phagosomal pH.

For rapid detection of phagosomal acidification, live macrophages were stained for 10 min with 250 nM LysoTracker Red DND-99 or LysoTracker Green DND-26 as appropriate. Stained cells were washed with serum-free RPMI 1640 lacking the probe and imaged immediately by live-cell fluorescence microscopy. When necessary, cells were pretreated with 1 µM ConA or 40 mM NH_4_Cl for 1 h prior to LysoTracker staining.

Ratiometric measurements of phagosomal pH were done using FITC-dextran according the work of Ohkuma and Poole (25) and following the protocol described by Canton and Grinstein (54). Additional details are provided in Text S1.

### Fluorescence microscopy.

Wide-field fluorescence microscopy was performed on a Leica DMI6000 B inverted microscope equipped with 40× (NA 1.3), 63× (NA 1.4), and 100× (NA 1.4) oil immersion PL-Apo objectives, a Leica 100-W Hg high-pressure light source, and a Photometrics Evolve 512 Delta EM-CCD camera. This microscope is also outfitted with an objective warmer and an enclosed heated stage insert with CO_2_ perfusion (Live Cell Instruments). This microscope is equipped with the ET-Sedat-quad 89000 series excitation and emission filter set (Chroma Technologies) for DAPI (4′,6-diamidino-2-phenylindole), GFP/FITC, Cy3/Alexa 555, and Cy5/Alexa 647 imaging. For live-cell imaging, coverslips carrying macrophages were placed in a magnetic imaging chamber and bathed in SF-RPMI buffered with Na bicarbonate and 25 mM HEPES. Live-cell imaging employed a heated stage and objective warmer set to 37°C and in the presence of 5% CO_2_ as necessary.

Images were acquired as z-series, and when appropriate, the raw data were deconvolved using the Leica LAS X software. This was done for LAMP-1 images, and the micrographs depicted represent the sum of the fluorescence from consecutive deconvolved z-slices. Images were contrast cropped, contrast enhanced linearly, and merged in ImageJ. The gamma was never altered, and fluorescence intensity measurements were only made on raw data.

### Murine systemic infection model.

Bacteria were cultured as previously described (50). Bacterial suspensions were diluted to an OD_600_ equal to ~0.3 (5.0 × 10^7^ CFU/ml) and used for injection. Six-week-old female BALB/c mice (Charles River Laboratories, Inc.) were injected via tail vein with 100 µl of bacterial suspension, and at 8 h postinfection, mice were anesthetized and then euthanized, and livers were harvested for plating. Extracted organs were placed into 3 ml of ice-cold 0.1% (vol/vol) Triton X-100 in 1× PBS and homogenized for 5 min by ballistic disruption in a Bullet Blender Storm (Next Advance, Troy, NY). Homogenates were serially diluted 10-fold and plated onto tryptic soy agar (TSA) for enumeration of the bacterial burden.

### Ethics statement.

Blood was obtained, with written permission, only from healthy adult volunteers, in compliance with protocol 109059 approved by the Office of Research Ethics at the University of Western Ontario. All animal protocols (protocol 2017-028) were reviewed and approved by the University of Western Ontario Animal Use Subcommittee, a subcommittee of the University Council on Animal Care. Protocols adhered to guidelines set out by the Canadian Council on Animal Care.

### Statistical analyses and software.

All statistical analyses were performed using GraphPad Prism software (San Diego, CA). When appropriate, one-way analysis of variance (ANOVA) and unpaired *t* tests were performed to determine statistical significance, with a *P* value cutoff of ≤0.05 to establish significance. Where appropriate, Bonferroni’s and Dunnett’s *post hoc* tests were performed to directly compare experimental means.
