# Supplementary figures and images for: Staphylococcus aureus Uses the GraXRS Regulatory System To Sense and Adapt to the Acidified Phagolysosome in Macrophages
Source: mBio. 2018 Jul 17;9(4):e01143-18. doi: 10.1128/mBio.01143-18 (PMC6050959; doi:10.1128/mBio.01143-18)

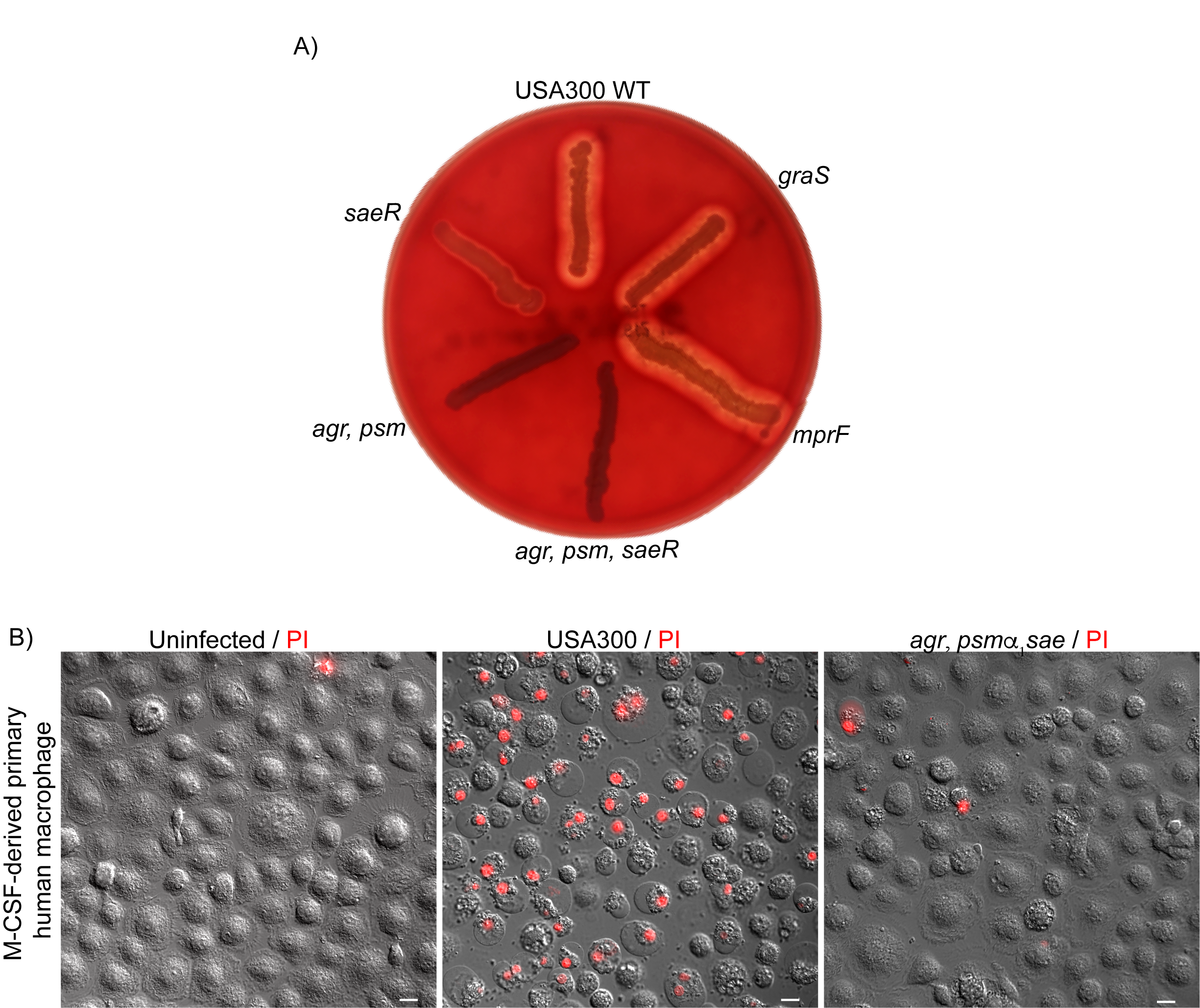

Supplement: FIG S1 [file mbo004183971sf1.tif]

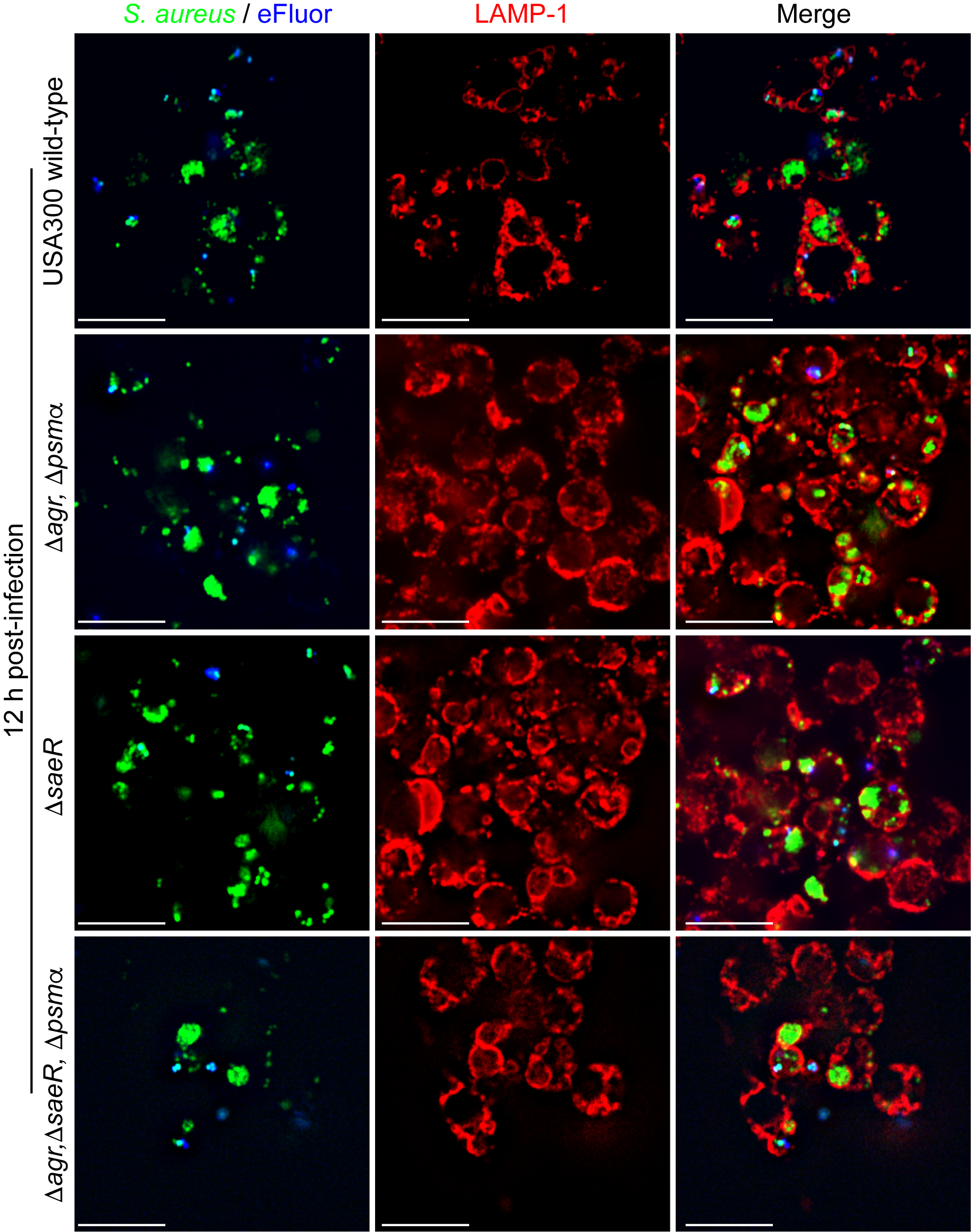

Supplement: FIG S2 [file mbo004183971sf2.tif]

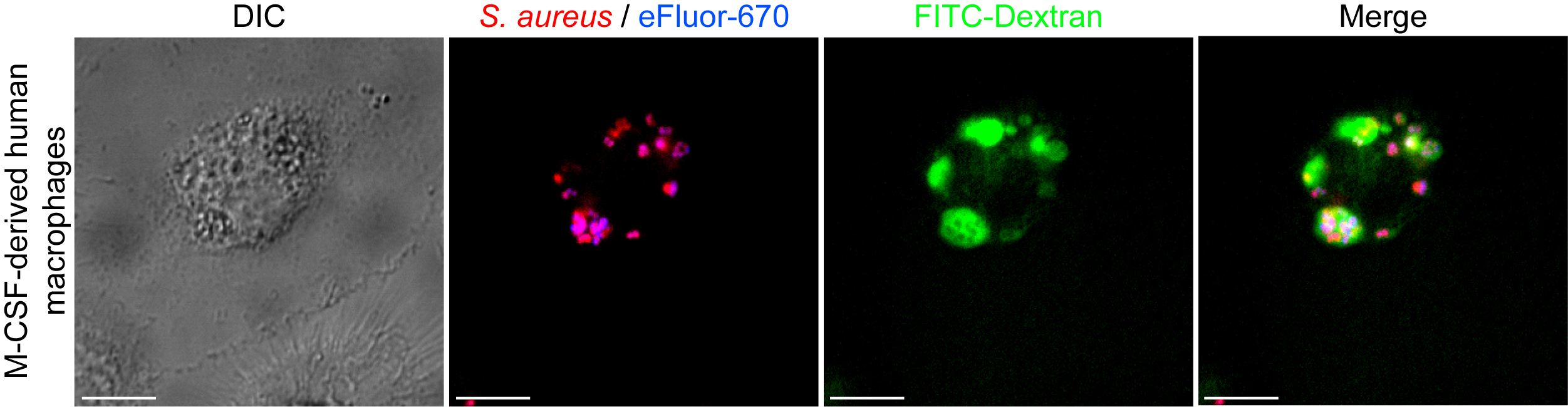

Supplement: FIG S3 [file mbo004183971sf3.tif]

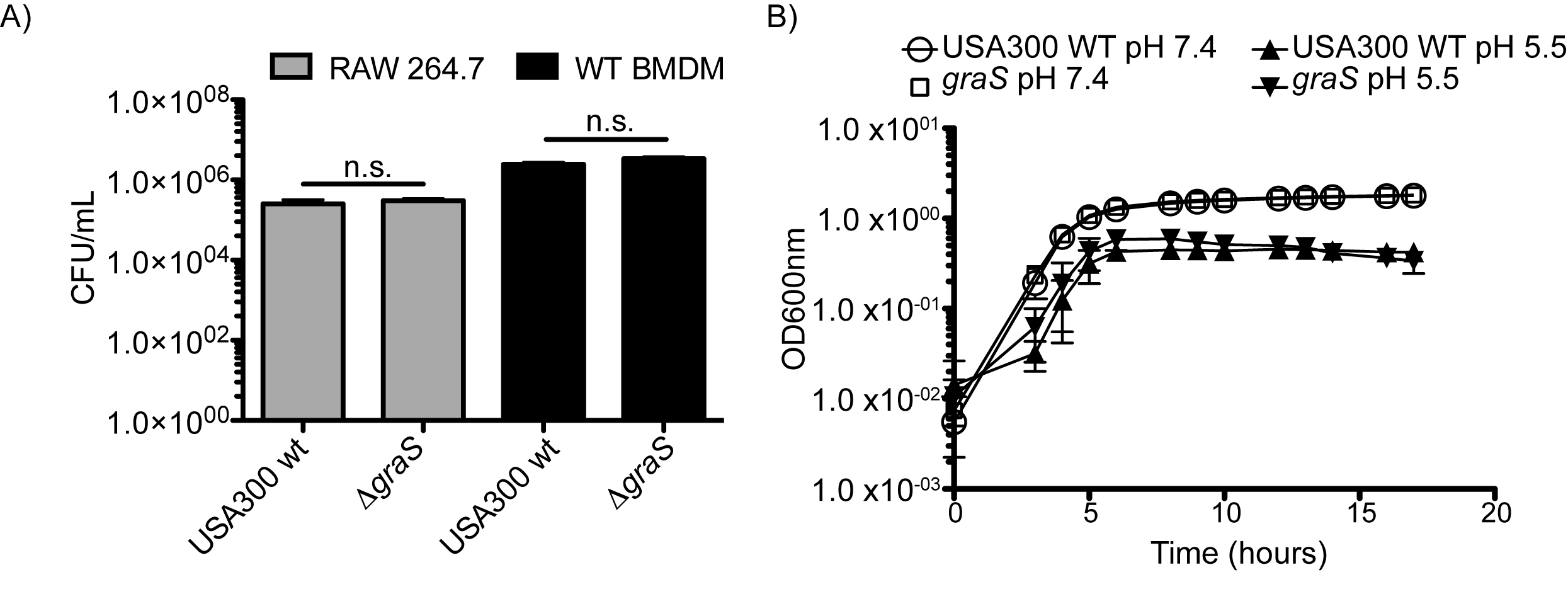

Supplement: FIG S4 [file mbo004183971sf4.tif]

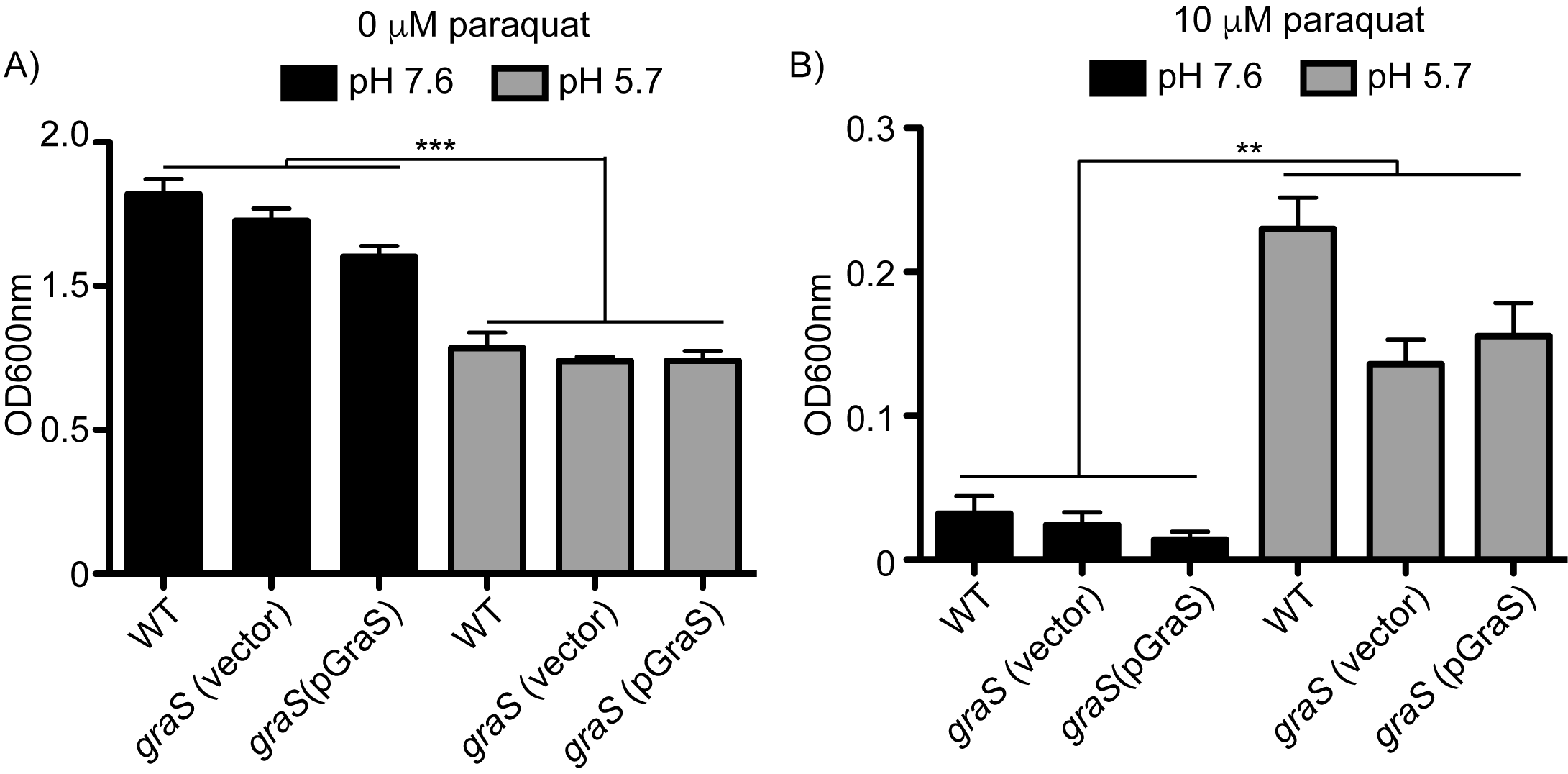

Supplement: FIG S5 [file mbo004183971sf5.tif]
