# Supplementary material for: Staphylococcus aureus Uses the GraXRS Regulatory System To Sense and Adapt to the Acidified Phagolysosome in Macrophages
Source: mBio. 2018 Jul 17;9(4):e01143-18. doi: 10.1128/mBio.01143-18 (PMC6050959; doi:10.1128/mBio.01143-18)
Supplement: TEXT S1 [file mbo004183971s1.docx]

**Supplementary methods**

**Generation of *S. aureus* mutants**

In brief the mutagenesis plasmids for deletion of the *apsS* gene and psmα genes were created by amplifying regions of homology from the chromosome of *S. aureus* USA300. The amplicons were digested with SacII and were ligated using T4 DNA ligase. Ligation products were then recombined into the parental pKOR plasmid using Gateway^®^ BP Clonase^®^ (Thermo Fisher Scientific). The resulting deletion plasmids pKOR::*psmα_1-4_* and pKOR::*apsS* were maintained in *E. coli* DH5α and passaged through *S. aureus* RN4220 at 30 °C before electroporation into *S. aureus* USA300. For the deletion of the *psm*α_1-4_ locus the primers 3’-psmAlpha-F (5’-ggggacaagtttgtacaaaaaagcaggctGTTGAATGAAGTATAATCATTT TTGAATTGTGG-3’) with 3’-psmAlpha-R (5’-TATATACCCGCGGTAAGATTACCTCCTTTGCTTATGAGTTAACTTC -3’) and 5’psmAlpha-R (5’-ggggaccactttgtacaagaaagctgggtTGCATA ACCTCCTTATTTCTAA TCTCTCGC-3’) with 5’-psmAlpha-F (5’-TATATACCGCGGTAATT TAAGCGAATTGAATA CTTAAAATTCTC-3’) were used to created regions of homology that flank the psmα genes. The SacII restriction sites are underlined. Confirmation of *psm*α_1-4_ deletion was done by PCR amplification using the primer pair 3’-psmAlpha-F with PSMalpha-5’ Flank-R (5’-GCAATGATAAGCGCAATAACAAACAGTGAAAATAG-3’) which anneals outside of the regions of homology used to create the deletion. These primers produce amplicons of 1.2 kbp and 1.6 kbp for the deletion mutant and wild-type bacteria respectively.

The deletion of *graS* was constructed similarly however primers *graS*-UP-SacII (5’– TTTGGTACCGTGCAATCGAAACAAAAG–3’) with *graS*-UPattB1 (5’– ggggacaagtttgtacaaaa aagcaggctAAGAATAAGGTGCGACCG–3’) and *graS*-DW-SacII (5’–GGACCTCCGCGGTAA ACATGCGTTTTGTTACTTAGAATTG–3’) with *graS*-DW-attB2 (5’–ggggaccactttgtacaaga aagctgggtAGCTTCCGACTTGTGAGC–3’) were used to amplify the desired regions of homology flanking the *graS* gene. Deletion of *graS* from the chromosome of *S. aureus* USA300 was confirmed the primers, *graS*-DEL-UP (5’–GCCATAAAAAGCCTCCAG–3’) and apsS-DEL-DW (5’– CTTTGATAGAACCTTCCGCTAC-3’).

To construct a double mutant lacking both the *psmα* genes and the *agr* quorum sensing locus phage transduction was performed using phage 80α. In brief, a phage lysate of *S. aureus* RN6911, an Agr null strain in which the *agr* operon is replaced with *tetM* (61) was created using standard protocols. The resulting phage 80α lysate was then used to transduce *agr::tetM* to *S. aureus* USA300 which was selected for by tetracycline resistance. Tetracycline resistant *S. aureus* colonies were then streaked onto TSA plates containing 5% (v/v) sheep blood to observe that the transductants were no longer hemolytic. Replacement of the wild-type *agr* locus with *tetM* was confirmed genetically by PCR using the primer pair AgrA-R (5’- GCTTACGAATTTC ACTGCCTAATTTG-3’) with AgrB-F (5’-CTTATCATCAAAGAGCCATTTGCCC-3’) which should only yield a product for wild-type bacteria. To further confirm site specific replacement of the *agr* locus with *tetM* the primers SAUSA300-1987-F (5’-CTTAAATGAAGTAGAACAGCAACGCG-3’) with TetM-5’out (5’-CTCCAATATTAATAATTTTCAT-3’) which anneal to the gene flanking the *agr* operon and the *tetM* gene respectively. To create the triple mutant lacking *agr*, *psmα *and* saeR* or to create the *saeR* mutant alone in our *S. aureus* USA300 the *saeR::* ::φNΣ was transduced from the strain NE1622 of the Nebraska transposon library into wild-type and the *agr*/*psma* strain using phage 80α and standard protocols. Disruption of *saeR* was confirmed by PCR analysis using primers flanking the *saeR* gene.

Disruption of *graS* in *S. aureus* Newman was done through transduction of the *graS* ::φNΣ, allele from the Nebraska transposon library using phage 80α. Verification of *graS* disruption was done by PCR analysis and by sensitivity to 64 μg/mL PmB at pH 5.5.

To generate an *S. aureus* Newman mutant which lacked the *ureABC* genes and had an *arcA* allele wild type Newman was mutated using pKOR as described above except EcoRI was used in place of SacII. For regions of homology to mediate targeted chromosomal recombination the following primer pairs, AttB1-Urease-5F (5’-GGGGACAAGTTTGTACAAAAAAGCAGGCTTCAAGCATCTACTTTGTCATG-3’) with Urease-5R (5’-TTTTTTGAATTCGCTCTCGTTGTGTAAAATGCA-3’) and Ureas-3F (TTTTTTGAATTCTAGGAGGAACATAGAATGATT-3’) with AttB2-Urease-3R (5’-GGGGACCACTTTGTACAAGAAAGCTGGGTCTATGATATGAAAATTCTAAC-3’) were used. Confirmation of the *ureABC* deletion was done by PCR using the following primer pair Ure-F (5’-CGCTGTATAGTTTAAGGTGATATTC-3’) with Ure-R (5’-CCAATTTTAATCGGATTTGCCAC-3’) which yield an amplicon of 2379 bp upon gene deletion and 4837 bp for wild-type bacteria. Upon confirmation of the *ureABC* deletion the *arcA*::φNΣ), allele was transduced from the Nebraska library Transposon Mutant (ID No. NE623) into the Δ*ureABC* mutant background using phage 80α. Confirmation of the *arcA* mutation was done by PCR using the primers ArcC-F (5’-CGCTGGTGAATCAAATAAGG-3’) AND Arc-Promoter-R (5’-CTTTATACAACATGTTTTTATGGGT-3’).

**Construction of the pALC2073::*apsS* complementation vector.** The *graS* gene was amplified from the *S. aureus* genome using PCR with the forward primer, graSUP (5’-TTTGGTACCGTG CAATCGAAACAAAAG–3’, and the reverse primer, graSDW (5’–TTTGGTACCCCATAATA GCAATAAACTCGCCTTC-3’), which incorporated *KpnI* cut sites (underlined) to each end of the amplified region. Empty pALC2073 and the *graS* amplicon were digested with *KpnI-HF* and ligated together. The resulting plasmid named pGraS.

**Fluorescence proliferation assay analysis of *graS* complementation.** *S. aureus* strains carrying either pALC2073 or pGraS were co-transformed with P_prsA_::*gfp*. For infections, each strain was cultured overnight in TSB containing 12 μg/mL chloramphenicol, 3 μg/mL erythromycin and 125 ng/mL anhydroustretracyline. Prior to infection cells were washed and labelled with eFluor-670 proliferation dye as described in the methods. Cells were washed and re-suspended in serum free RPMI for infection of RAW macrophages at an MOI of 10. At either 1.5 or 12 h post-infection macrophages were fixed and immunostained for LAMP-1 as described in the methods section. To quantify the fraction of macrophages containing replicating bacteria the total number of macrophages containing eFluor-negative yet GFP-positive bacteria were counted for each strain and then normalized to wild-type *S. aureus* USA300 which was set at 1.

**Construction of the pGYlux::*mprF* reporter plasmid.** The *mprF* promoter was cloned directionally into the into pGylux plasmid. In brief the *mprF* promoter was PCR amplified using the following primer pair : mprF::lux-F: CCA ATT ATT TAA ACG TCG ATA CAA T**GG ATC C**TA TGG TAA TGA TGT AGG TG and mprF::lux-R: GAA TAA CAA GCG ATG CCC **GTC GAC** CAA TAA ATA GTA ACA CTA AGG. The resulting amplicon was digested with BamHI and SalI and cloned into pGYlux that was similarly digested. The clone was identified by PCR and confirmed by DNA sequence analysis.

**Ratiometric pH measurements**

Imaging of FITC fluorescence was done by live cell imaging of FITC-dextran loaded macrophages infected with live and dead *S. aureus* USA300 expressing mCherry. Imaging of FITC fluorescence was done by sequential excitation at 434 nm and 490 nm using an ultrafast excitation filter wheel controlled by the Leica LAS X imaging software. Emitted FITC fluorescence was passed through a 504 nm emission filter prior to detection by a Photometrics Evolve 512 Delta EM-CCD camera. To determine the FITC fluorescence ratio the background-corrected FITC emission at ex490 nm was divided by the background corrected FITC emission at ex434 nm. To determine the pH that this ratio corresponds to, *in situ* pH clamping of FITC-dextran loaded cells was done at the end of each experiment. To this end live macrophages were incubated with K^+^-rich buffers containing nigericin (140 mM KCl, 1 mM MgCl_2_, 1 mM CaCl_2_, 5 mM glucose with appropriate buffer at pH 7.5 through 4.0 at 0.5 pH unit increments. The resulting background corrected FITC fluorescence ratios were plotted versus pH and fitted to a Boltzman sigmoid which was used to interpolate ratios measured from phagosomes containing *S. aureus*.
